# Integrated Hemicellulose Extraction and Papermaking Fiber Production from Agro-Waste Biomass

**DOI:** 10.3390/polym15234597

**Published:** 2023-12-01

**Authors:** Adrian Cătălin Puițel, Cătălin Dumitrel Balan, Gabriela-Liliana Ailiesei, Elena Niculina Drăgoi, Mircea Teodor Nechita

**Affiliations:** 1 Faculty of Chemical Engineering and Environmental Protection “Cristofor Simionescu”, “Gheorghe Asachi” Technical University Iasi, Bd. Prof. Dimitrie Mangeron, No. 73, 700050 Iasi, Romania; adrian-catalin.puitel@academic.tuiasi.ro (A.C.P.); catalin-dumitrel.balan@academic.tuiasi.ro (C.D.B.); elena-niculina.dragoi@academic.tuiasi.ro (E.N.D.); 2 “Petru Poni” Institute of Macromolecular Chemistry, 41A Grigore Ghica Voda Alley, 700487 Iasi, Romania; gdarvaru@icmpp.ro

**Keywords:** corn stalks, xylan HC, papermaking pulp, hot alkaline extraction, tensile index, burst index

## Abstract

The present study deals with the valorization of corn stalks in an integrated processing strategy targeting two products: extracted hemicelluloses (HC) and papermaking fibers. Preliminary trials were conducted to assess the individual or the combined effects of biomass treatment on the quality of the obtained hemicelluloses and papermaking fibers. Depending on the hot alkaline extraction (HAE) conditions, the extracted HC had a xylan content between 44–63%. The xylan removal yield ranged between 19–35%. The recovery of HC from the extraction liquor and final black liquor was significantly affected by process conditions. The experimental approach continued with the study of HAE conditions on the obtained paper’s mechanical properties. The optimization approach considered conserving paper strength properties while achieving an equilibrium with the highest possible HC extraction yield. The optimal values are sodium hydroxide concentration (1%), process time (33 min), and temperature (100 °C). The xylan content in the separated HC sample was ~55%. An extended extraction of HC from the resulting pulp under hot alkaline conditions with 5% NaOH was performed to prove the HC influence on paper strength. The xylan content in HC samples was 65%. The consequence of xylan content reduction in pulp leads to 30–50% mechanical strength loss.

## 1. Introduction

Developed at the dawn of this century, the Circular Economy (CE) [1,2,3] and Zero Waste (ZW) [4,5,6,7] are two particularly attractive and popular management concepts that can be easily implemented in agriculture to complete the relatively “mature” concepts of Integrated Farming System (IFS) [8,9] and Integrated Crop Management (ICM) [10,11]. The conversion of agro-waste into value-added products meets the requirements of all of these management concepts. There are strong arguments that in the near future, this will be an environmentally friendly activity and a profitable business venture [12,13,14].

There is an inherent relationship between global population growth and food demand, and agriculture must develop to meet these needs. Although agricultural development does not necessarily imply an increase in agro-waste production, forecasting major agricultural products such as grains (maize, wheat, rice) indicates consistent growth. Consequently, various straws and stalks that are sought after as raw materials and primary resources will be readily available. Data Bridge Market Research shows that the wheat straw market was valued at USD 643.6 million in 2021 and is expected to reach USD 1330.24 million by 2029 [15]. The production of corn (maize) is even higher; according to Statista, the worldwide production of grain in 2022/23 places corn first with an estimated production of 1151.36 million metric tonnes, followed by wheat with 783.8 and rice with 502.98 million metric tonnes [16]. In Europe, wheat comes first in 2023, with an estimated production of 143.2 million tonnes, and corn second with 64.5 million tonnes [17]. Romania ranks fourth among corn-producing countries in the European Union, trailing Ukraine, France, and Italy [18]. 

The corn agro-wastes are typically made up of leaves (20%), husks (8%), cobs (14%), stalks (42%), and other components (16%) [19]. The stalk is the main component of the corn plant, and this, along with the volume of corn produced, places corn stalks (CS) among the most prevalent agricultural waste worldwide. Following the various management recommendations (CE, ZW, IFS, ICM), many different initiatives were made to increase the value of this abundant and renewable agro-waste. The traditional “classic” valorization methods mostly recommended by ICM and IFS management strategies include the use of CS to prepare animal feed [20], as fuel [21], or as soil fertilizer [22]. The opportunities for CS valorization are expanded by the contemporary management strategies (CE, ZW). Nowadays, CS residues may be recycled to produce cellulose/epoxy resin composites [23], cellulose nanofibrils [24], cellulose composites [25], carbonaceous composite adsorbents [19], levulinic acid and biocarbon electrode material [26], second-generation bioethanol [27], biomethane [28], chemical pulp [29] and various biomaterials (plastics, hydrogels, fibers, composites) [30]. The use of agricultural waste biomass as fillers in different bio-composites [31], bio-plastics, tires [32], and other reinforced polymers [33,34] is one of the most promising directions to its conversion into value-added goods [34]. The CS fibers in particular were tested as fillers for various composite materials such as: tire rubber powder composite [35], polylactide composite [36], colorless and odorless bio-plastics [37] and other green composites [38]. Moreover, various integrated biorefinery processes were proposed to fully convert CS into value-added products [30,39,40]. A few examples include the coproduction of: saccharides, pulp, and lignosulfonate [41]; biodegradable film, bioethanol, and soda pulp [42]; fermented liquid feed and biologically modified biochar [43]; tissue paper and glucose [44]; hemicellulose and ethanol [45]; ethanol, furfural, and lignin [46]; ethanol and L-lactic acid [47]; hemicellulose, lignin, and activated carbon [48]; hemicellulose, lignin, cellulose (further processed to paper) [49]; cellulose, lignin, and xylose [50]; micro/nano-cellulose fibers, monosaccharides, and lignin fractions [51]. 

Such an ambitious level of corn stalk utilization (complete) to such a wide range of products necessitates a wide range of methods and/or combinations of methods. Some of the techniques are classic, e.g., alkaline extraction [52] or enzymatic hydrolysis [53,54,55], in some cases improved with complementary physicochemical pretreatments such as P-toluene sulfonic acid [54], mild alkaline presoaking (Na_2_S) and Organosolv [55], ammonia [28] microwaves [53], ultrasound [51] and freeze/thaw cycles [28]. Combinations of processes are frequently used to fully transform corn stalks into finished goods or useful intermediates. To achieve the required yield and selectivity, most technological approaches necessitate the adjustment of specific parameters (e.g., treatment time, temperature, pH, solvent mixture) and process optimization. A few examples of technological processes include one-step aqueous formic acid fractionation [49]; two-stage fermentation (*S. cerevisiae* and *B. coagulans*) [47]; solvent extraction (toluene/ethanol) followed by NaClO_2_/acetic acid delignification and NaOH pulping [24]; hot water, alkali (NaOH), and modified alkali (NaOH and NaBH_4_) [42]; pre-hydrolysis (H_2_SO_4_) and alkaline sulfite cooking [41]; anaerobic fermentation (rumen inoculum) and pyrolysis [43]; hydrothermal pretreatment and batch acid hydrolysis [26]; peracetic acid and maleic acid pretreatment and enzymatic hydrolysis [50]. 

The current investigation focuses on coproducing hemicellulose polysaccharides and pulp using corn stalks as raw material. This study follows: (i) the identification of the appropriate coproduction process and (ii) the optimization of hemicellulose extraction through this process in a way that prevents the loss of specific paper qualities (tensile index and burst index). Screening trials were conducted in order to develop the optimal coproduction procedure. Various sequences of hot water treatment (HWT), soda pulping (SP), and hot alkaline extraction (HAE) were tested during trials. Treatment time, temperature, and alkali concentration were the monitored process parameters during the trials and optimization stage. Hemicelluloses were separated and characterized after each technical stage. The controlled extraction of hemicelluloses aims at preserving the pulp and, consequently, paper mechanical characteristics—tensile and burst indexes. The novelty of the work is represented by the technical approach combined with the optimization technique that establishes a successful method for producing acceptable yields of hemicellulose and paper with satisfactory mechanical properties.

## 2. Materials and Methods

### 2.1. Materials

Corn stalks (a common Zea Mays hybrid) were provided free of charge by Romanian farmers. After harvesting, the CS were dried at room temperature to 8% humidity. The CS were then crushed and sieved to 0.2 mm particles per the standard chemical analysis technique. The CS were cut into 50 mm pieces for hemicelullose (HC) extraction and/or pulping studies. 

All chemicals and reagents used are of analytical purity. Solutions of 99% purity of cellobiose, glucose, xylose, galactose, and arabinose, provided by Flucka, were used to obtain the calibration curves in the concentrations range of 0.1–1 g/L. Purified xylan extracted from beechwood was purchased from Sigma Aldrich (X4252 10G; St. Louis, MO, USA) and was used as reference material.

### 2.2. Equipment

#### 2.2.1. Laboratory Reactor and Pulp Processing Equipment 

The extraction and pulping experiments were conducted in a 10 L stainless steel laboratory rotating digester that was electrically heated and equipped with a temperature controller. The obtained pulps were refined at different revolutions in a Jokro mill, following the procedure described by Danielewicz et al. [56]. The refined and non-refined pulps were converted into paper sheets on a Rapid Koethen laboratory sheet former ISO 5269/2 [57]. The testing of the mechanical strength properties (tensile strength and burst strength) and necessary calculations were performed according to ISO 1924-2:2008 [58] and ISO 2758:2014 [59] using a Zwick Roell Z0.5 testing machine (ZwickRoell GmbH & Co. KG Headquarter, Ulm, Germany). 

#### 2.2.2. HPLC Analysis

A Shimadzu Nexera LC 40D liquid chromatography system equipped with a Shodex SP0810 column (300 × 8 mm, particle size 7 µm) heated at 65 °C was employed to perform the required HPLC analysis. The refractive index detector (Shimadzu RID 20A, Kyoto, Japan) was set at 40 °C. The flow rate of the mobile phase (ultrapure water) was 0.6 mL/minute. The injection volume was set between 20 to 40 µL to accommodate sugar concentrations. Each sample and standard solution (containing cellobiose, glucose, xylose, galactose, and arabinose of analytical grade) was filtered before injection using 0.2 μm syringe PTFE filters.

#### 2.2.3. Spectroscopy

The FTIR spectra of selected hemicellulose samples were recorded by Agilent Cary 630 (Santa Clara, CA, USA) using the potassium bromide pellets technique on disks containing finely ground samples at 1% content.

The 1H NMR spectroscopy data were obtained by dissolving 16 mg of HC samples in 0.65 mL deuterated water and then pipetted into NMR tubes. Spectra were recorded on a Bruker Avance NEO 400 MHz spectrometer (Billerica, MA, USA), operating at 400.1 MHz for 1H nuclei, with a 5 mm four nuclei direct detection z-gradient probe using standard pulse sequences, as delivered by Bruker with TopSpin 4.0.8 spectrometer control and processing software (version 4.0 8). Chemical shifts are reported in δ units (ppm) and were referenced to the residual solvent signal at 4.7 ppm. 128 scans were used for spectra registration. 

#### 2.2.4. Other Equipment

Sorvall GLC2 centrifuge equipped with an HL-4 rotor, CF value-2012, 3000 r.p.m. was used for precipitate separation (lignin, respectively HC). 

Ubbelohde and capillary (glass with jacket, PSL-Rheotek type C) viscometers were used to determine the efflux time of pure solvent, HC, and cellulose solutions. 

### 2.3. Experimental Approach

**Figure 1:**
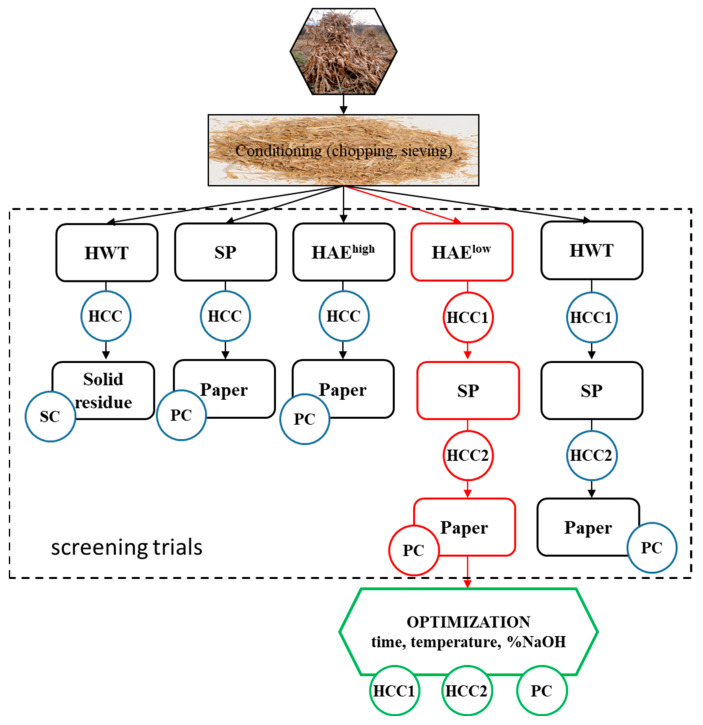
The experimental approach. (SC = solid residue characterization; PC = pulp and paper characterization; HCC = hemicellulose characterization; 1 = first extraction; 2 = second extraction).

Screening trials (Figure 1) were utilized to determine the best strategy for achieving high extraction yields in HC and pulp, which was then used to manufacture paper with satisfactory mechanical qualities. The procedures used for HC extraction and pulp production were hot water treatment (HWT), soda pulping (SP), and hot alkaline extraction at low (HAE^low^) and high (HAE^high^) alkali charges. The selected sequence was further optimized considering time, temperature, and alkali concentration as process variables. The optimization goal was not to maximize the HC extraction yield but to produce an extraction yield that preserved the pulp qualities (including HC content) to prepare paper sheets with satisfactory mechanical properties (tensile and burst indexes). 

All experiments were performed in triplicates unless otherwise stated by the mentioned standard methods. The accepted maximum relative standard deviation value was less than 5%.

The materials recovered from each experimental method (HWT, SP, or HAE), whether solid or liquid (extraction liquor, black liquor), were analyzed for HC, cellulose, and lignin content. 

### 2.4. Extraction Methodology

#### 2.4.1. HWT Treatment 

Typical HWT experiments use 300 g of oven-dried (o.d.) corn stalks treated in the laboratory reactor at a solid-to-liquid ratio of 1:10. The treatment lasted 60 min at 100 °C. The heating time was 20 min. At the end of the HWT process, the resulting liquid phase was saved for further characterization and HC separation. The HWT-treated corn stalks were washed, dried to a suitable moisture content (8–10%), and then used for soda pulping. The material loss was determined gravimetrically.

#### 2.4.2. Soda Pulping

The control soda pulping process (using untreated CS) was carried out in the same reactor (as HWT) under the following conditions: solid-to-liquid ratio of 1:20; heating time of 20 min; and cooking time of 30 min at 140 °C. The pressure during pulping was kept constant at 0.2 MPa. The active alkali charge was 12% expressed as NaOH units. The corresponding white liquor had a 0.6% NaOH concentration. The HWT followed by SP (Figure 1) treatments were performed in the same conditions as control pulping (SP). The obtained pulps were washed and used for further analysis and sheet formation. The solid yield was determined by gravimetric means.

The SP processes that followed HAE^high^ and HAE^low^ during trials were continued in the same reactor by increasing the temperature to 140 °C. The pulping stage was set for 30 min. After the pulping timer ran out, the heating was turned off, and the solid and liquid phases (black liquor) were separated and processed separately. 

#### 2.4.3. Hot Alkaline Extractions of Corn Stalks

In the trial phase of this study, the hot alkali extraction of hemicelluloses was performed using two different experimental approaches: high alkali charge (HAE^high^) and low alkali charge (HAE^low^). After that, an optimized approach was identified and conducted (HAE^opt^).

HAE^high^ involves extracting hemicelluloses from corn stalks with solutions containing 5% NaOH and 3% NaOH, respectively. In both experiments, CS (200 g o.d.) were immersed in NaOH solutions at a solid-to-liquid ratio of 1:20. The reactor was closed and heated to 100 °C. The extraction time was set to 60 min. After the extraction, the liquid phase (HAE^high^ liquor) and the remaining solid material were separated, characterized, and/or further processed. 

HAE^low^ was conducted for 60 min at 100 °C using a 0.6% NaOH solution. Following treatment, samples of HAE^low^ liquor were extracted for further hemicellulose separation. 

HAE^opt^—an extended hemicellulose extraction from pulp was performed at optimal process parameters. The following conditions were used for this hot caustic extraction (HCE): 5% NaOH concentration, solid-to-liquid ratio 1:10, temperature of 100 °C, and treatment time of 60 min. The recovered liquor was then processed for hemicellulose separation using the procedure described in Section 2.4.4. 

#### 2.4.4. Separation and Purification of HC from WHT, HAE, and SP Liquors

The ethanol precipitation method was used to separate hemicellulose from the liquors obtained during the experimental procedures. In brief, 50 mL liquor samples were neutralized to pH 4.5 with acetic acid. A first centrifugation stage was performed to remove the precipitated lignin. The supernatant was then mixed with 2 volumes of analytic purity ethanol (96%) and stored at −18 °C for 60 min. The precipitated HCs were separated by centrifugation at 3000 r.p.m. for 10 min. Next, two rounds of ethanol washing were performed. Following each washing, a 5-min centrifugation was carried out at 3000 r.p.m to separate the solid from the ethanol. The crude HC samples were dried at 50 °C before further investigation. 

### 2.5. Characterization Methods

#### 2.5.1. CS, Treated CS, and Pulp Chemical Characterization

Several analytical procedures were used to determine the chemical composition of raw CS in terms of both major (polysaccharides and lignin) and minor components: ash–TAPPI T 211 om-02, 2002 [60]; hot water extractives—TAPPI T 207 om-88 [61]; organic solvent extractives T 204 cm-97 [62]; acetone extractives (AE)—TAPPI T280 pm-99 standard (2000) [63]. While acid-insoluble lignin (AIL) and acid-soluble lignin (ASL) were determined using the sulfuric acid two stages hydrolysis method specified by NREL/TP-510-42618 method [64], the major polysaccharide components (cellulose and hemicelluloses) of the biomass and the obtained papermaking fiber were determined following an adapted procedure of that described by Sluiter et al. [65]. The adaptation involved neutralizing the hydrolysate from a G3 crucible filter to pH 5.6 before HPLC analysis. 

#### 2.5.2. HC Characterization 

The carbohydrates present in liquor samples were analyzed after they were treated with 4% sulfuric acid (60 min at 121 °C) according to NREL (LAP) TP-510-42623 [66]. Samples of 60 to 80 mg were suspended in 5 mL of 1 M NaOH and vigorously shaken for at least 30 min to facilitate dissolution. The complete hydrolysis was achieved by treating the samples with 4% sulfuric acid for 60 min at 121 °C. The acid treatment completes the hydrolysis of the polymeric carbohydrates extracted during HAE. Following hydrolysis, the samples were neutralized, and the concentration of monosaccharides was determined using HPLC. 

#### 2.5.3. HC Recovery Yield, Solid Extraction Yield

The glucan, xylan, and arabinan content of the liquor were added to determine the total amount of polysaccharides (PStot). The conversion of monomer concentrations to their corresponding polymer concentrations was realized considering the ratio between the molecular weight of the anhydro-sugar unit and sugar unit (162/180 = 0.9 for C6 sugars and 132/150 for C5 sugars).

The HC recovery yield (HCRY) was then calculated using Equation (1) as the ratio between the number of polysaccharides in recovered crude HC (o.d.) and the number of polysaccharides theoretically determined in either extraction or black liquor.

(1) $$HCRY\left(\%\right)=\frac{\sum MS_{i}\left(\%\right)\cdot m_{HC}}{\sum C_{MS_{i}}\cdot V}\times 100$$

where *MS_i_* (%) is the content of individual polysaccharides (glucan, xilan, arabinan) content in the *HC* sample; m_HC_ is the mass of the *HC* sample, in g; *C_MSi_* is the concentration of the individual sugar in the analyzed sample, in g/L; *V* is the volume of liquor sample, in L.

The solid extraction yield (SY, %) was calculated using Equation (2).

(2) $$\mathrm{SY}\;\left(\%\right)=\frac{m_{f}}{m_{i.}}\times 100,$$

where SY (%) represents the solid yield; m_i_ is the o.d. weight of the initial CS biomass; m_i_. is the o.d. weight of the CS after the treatment or treatment sequence.

#### 2.5.4. Polymerization Degree (DP) of Obtained HC and Pulp 

The degree of polymerization of HC was proven by using viscosity data as described in literature [67]. In brief, the samples were dissolved in a 0.04 M cupriethylenediamine (CED) solution, and the intrinsic viscosity [η] was determined at 25 °C. The Staudinger–Mark–Houwink equation of xylan in CED is (Equation (3)) [67,68,69]:

(3) $$\left[\eta \right]=2.2\times 10^{-2}\times DP^{0.72}$$

The obtained pulps’ *DP* was established after determining the intrinsic viscosity (Equation (4)) in 0.5 M CED solution [70].

(4) $$\left[\eta \right]=2.28\times DP^{0.76}$$

#### 2.5.5. Pulp Refining and Laboratory Paper Strength 

Following washing and refining (beating), the obtained pulps were transformed into paper sheets that were subjected to analysis of tensile strength (ISO 1924:2008) [58] and burst strength (ISO 2758:2014) [59]. 

#### 2.5.6. The Severity Factor

The temperature and duration of extraction can be combined in a single parameter, the severity factor (*SF*), to reduce the total number of experiments. The *SF* is defined as the combination of extraction time and the temperature (Equation (5)) [71,72].

(5) $$SF=\log_{10}(\tau \times e^{\frac{T-100}{14.75}})$$

where τ is the processing time at selected temperature *T*.

### 2.6. Optimization Procedure

The results obtained in the trial phase indicate that the sequence HAElow–SP–Paper (Figure 1) generates the best equilibrium between HCRY and paper strength. Therefore, this sequence was selected for further optimization. Response surface methodology (RSM) was selected as an optimization procedure for modeling the HC extraction. The independent variable parameters and their variation range (Table 1) were chosen based on previous experience [52,73]. 

The model dependent variables were: Y1—xylan content in the recovered hemicelluloses (XHC, %); Y2—xylan removed from corn stalk biomass XRCS, (%); Y3—tensile index of resulting paper sheets (TI, N·m/g); Y4—burst index of obtained paper sheets (BI, kPa·m^2^/g). Experimental design and data processing were performed by using Stat-Ease Design-Expert Software (version 7). The experimental data were then used to reveal the equations describing the relationship between selected process parameters and model responses. 

## 3. Results

### 3.1. Chemical Composition of CS and HWT CS Solid Residue

According to the literature analysis, a wide range of factors influence the chemical composition of CS (Table 2), including corn variety, precipitation/irrigation level, fertilization, harvesting period, harvesting equipment, and others. These factors affect not only the corn quality but also the HC, cellulose, lignin, and ash content of the CS (Table 2). A relatively mild treatment such as HWT only slightly affects the chemical composition of the CS. Aside from xylan, which is partially extractable with hot water, the other polysaccharide content appears slightly increased due to CS weight loss after HWT treatment (Table 2). Kim and Lee also reported HWT selectivity for xylan extraction and glucan stability in such treatment [74]. 

### 3.2. Solid Residue and Pulp Composition—Trial Results 

The raw CS soda pulping process generates the maximum solid yield. This is most likely caused by the SP process’s lower alkali charge than other treatment procedures/sequences. The HAE^low^–SP treatment sequence comes in second place, with a slightly lower SY value (Table 3). 

The SY value, in conjunction with the polymerization degree (Table 4), directly impacts the paper’s qualities in terms of the tensile and burst index values. The increase in C_NaOH_ (HAE^high^, 3%NaOH; HAE^high^, 5%NaOH) reduces both the HC and lignin content of the pulp and/or solid residue. The effects of light pretreatments (HAE^low^, HWT) carried out before SP on the SY are minor (Table 3). Yet, the paper’s resistance qualities are acceptable for the HAE^low^–SP treatment sequence (Table 4).

### 3.3. Liquor’s Chemical Composition (% wt.)—Trial Results

Samples of the liquid phase were collected after each individual and or sequential CS treatment. Black liquor (BL) refers to the liquid phase acquired after soda pulping, while extraction liquor (EL) refers to the liquid phase acquired after each primary treatment.

The amount of extracted xylan directly correlates with the NaOH concentration in the liquid; the higher the concentration, the higher the xylan content (Table 5). However, the cellulose produced under these circumstances (high alkali charge) has weaker resistance properties and lower DP than cellulose obtained at low alkali charge (HAE^low^, EL). It is worth noting that the DP increases as the NaOH concentration increases (Table 5). The EL produced in the absence of alkali (HWT) has a relatively low amount of xylan (7.7%) and a usually low amount of sugars, primarily glucan oligosaccharides residue. The EL produced at a low alkali charge (HAE^low^) gives a satisfactory level of xylan extraction (49.06%) that can be further increased up to 61.82 by complementary SP treatment (HAE^low^–SP). The chemical analysis of the reference material (xylan from beechwood) was also performed, and the results in terms of DP are very close to those obtained for the HAE^low^–SP sequence (255 vs. 262). 

Since the sequence HAE^low^–SP allows the simultaneous production of two commodities (paper pulp and HC–xylan) with good resistance properties (Table 4) and acceptable extraction yields (Table 5), this study was further focused on the optimization of this particular extraction procedure. 

### 3.4. Optimization of HAE Parameters: Influence on HC Xylan Content, Xylan Removal Yields, and Pulp Properties

Table 6 shows experimental conditions (experiments programmed using the central composite design) and their associated results in terms of: (i) xylan content of crude o.d. hemicellulose samples recovered from extraction liquor (XHC); (ii) xylan extraction yield from the CS (XRCS). The XRCS (%) values were computed according to Equation (6); (iii) paper tensile index (N·m/g) and (iv) paper burst index (kPa·m^2^/g) (obtained after beating at 1100 rpm)

(6) $$\mathrm{XRCS}\;\left(\%\right)=\frac{C_{xliq}\cdot V_{liq}}{X\left(\%\right)\cdot m_{CS}}\times 100$$

where *C_xliq_* is the concentration of xylan determined in the HAE liquor by HPLC in g/L; *V_liq_* is the volume of the liquor existing in the reactor at a specific moment of the extraction, in L; *X* (%) is the xylan content of the *CS*; *m_CS_* represents the o.d. weight of a working *CS* sample, usually 200 g.

Only two experiments (no. 11 and 21 from Table 6) resulted in HC with a xylan content greater than 60%. However, because the study’s goal was not to achieve the highest HC extraction yields, but to generate an option that did not interfere significantly with paper strength properties, the extraction parameter values provided by the experiments discussed cannot be considered optimal. 

The Equation (7) relates the independent variables: *X*_1_, *X*_2_, *X*_3_ (Table 1) to system responses: *Y*_1_—XHC (%); *Y*_2_—XRCS (%); *Y*_3_—TI, (N·m/g); and Y_4_—BI (kPa·m^2^/g). The terms *β*_0_, *β_i_*, *β_ij_*, *β_ii_* represent the equation constants. Table 7 presents the equation coefficients and statistical parameters.

(7) $$Y_{1-4}=\beta_{0}+\overset{3}{\underset{i=1}{\sum }}\beta_{i}X_{i}+\overset{3}{\underset{\begin{array}{c} i=1\\ j=1 \end{array}}{\sum }}\beta_{ij}X_{ij}+\overset{3}{\underset{i=1}{\sum }}\beta_{ii}X_{i}^{2}$$

**Figure 2:**
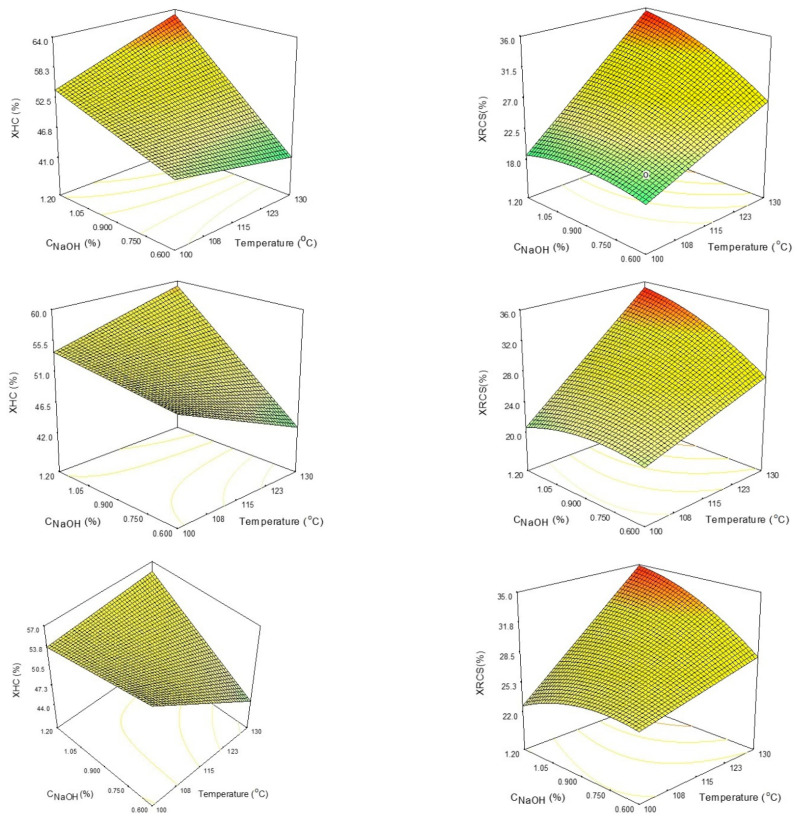
The influence of process parameters (temperature and C_NaOH_) towards XHC% (**left column**) and XRCS% (**right column**) after 30 min (1st row), 60 min (2nd row), and 90 min (3rd row). The color ranges from green (low values) to yellow and orange (medium values) to red (high values).

The evolution of XHC% and XHCS% as a function of temperature and NaOH concentration for constant time (30, 60, and 90 min) is shown in Figure 2. A first-order polynomial describes how the xylan content of the separated hemicelluloses depends on the process parameters. The simultaneous rise in temperature and C_NaOH_ caused a considerable increase in XHC. The XHC is barely impacted by lengthening the course of treatment.

A second-order polynomial equation describes the xylan removal from corn stalk biomass dependence on process parameters (Figure 2). It can be observed that the C_NaOH_ is the most important factor in the process, followed by temperature and time. The extraction yield can be increased by increasing C_NaOH_ and/or temperature. Extending the course of treatment has little effect on the XRCS.

**Figure 3:**
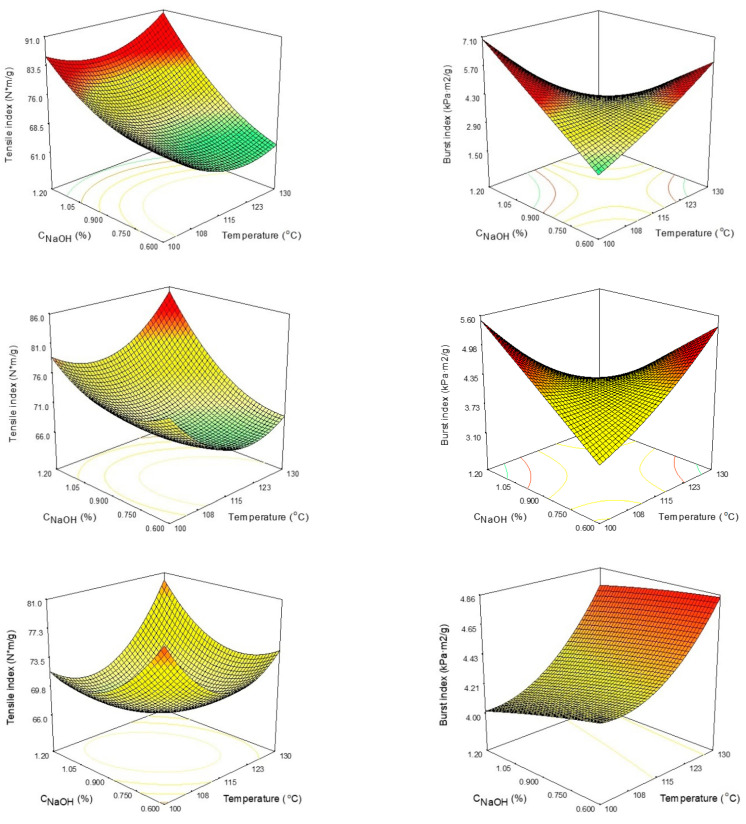
The influence of process parameters (temperature and C_NaOH_) towards tensile index (**left column**) and burst index (**right column**) after 30 min (1st row), 60 min (2nd row), and 90 min (3rd row). The color ranges from green (low values) to yellow and orange (medium values) to red (high values).

Produced pulp paper sheets’ TI is greatly affected by the sodium hydroxide content (Figure 3). TI increases with C_NaOH_ and decreases with increased treatment time. The combined action of sodium hydroxide and temperature has a positive effect; however, the cumulative interaction of C_NaOH_ and treatment time has a negative effect, decreasing TI. BI is negatively affected by the simultaneous increase in temperature and C_NaOH_ (Figure 3). Also, an increase in process time is accompanied by a BI decrease.

The ANOVA analysis of the proposed models is presented in Tables S1–S4, which are included in the Supplementary Material. 

The optimum hot alkaline extraction (HAE^opt^) process parameters, considering maximizing HC extraction yield while maintaining the papermaking properties, are temperature = 100 °C; sodium hydroxide concentration = 1 percent (wt.); and extraction time = 33 min. Table 8 illustrates the experimental confirmation of the predicted values by the proposed mathematical model. 

### 3.5. Complementary Extraction Treatments—Hot Caustic Extraction: Influence of HC Content on Refinability and Strength Properties of Paper Sheets

This experimental phase aimed to determine the impact of HC refinability and refining degree on the paper’s mechanical strength characteristics. Therefore, the results of the HAE^opt^ were further compared with a more aggressive extraction treatment, hot caustic extraction (HCE), expected to produce higher HC extraction yields (and, consequently, a pulp with lower HC content). To highlight the contribution of the HC content in pulp on the mechanical strength properties of paper, the TI and BI of the paper produced after HAE^opt^ and HCE treatments were compared. Based on the experimental results from the trial phase and optimization stage, the selected HCE process parameters were temperature = 100 °C, sodium hydroxide concentration = 5% (wt.), and extraction time = 60 min. The solid–liquid ratio was 1:20. 

Table 9 shows the chemical contents of the pulps obtained after HAE^opt^ and HCE extractions. The HCE treatment reduced the amount of xylan by around 50%. 

**Figure 4:**
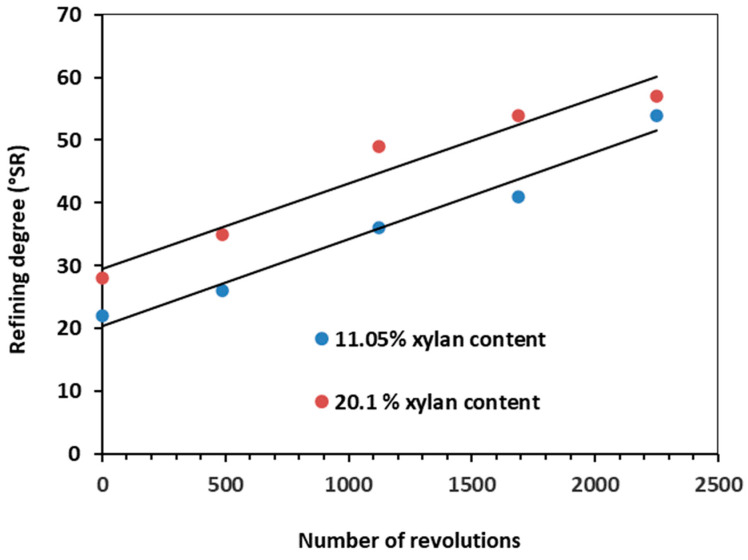
Evolution of the refining degree as a function of the refining input energy.

**Figure 5:**
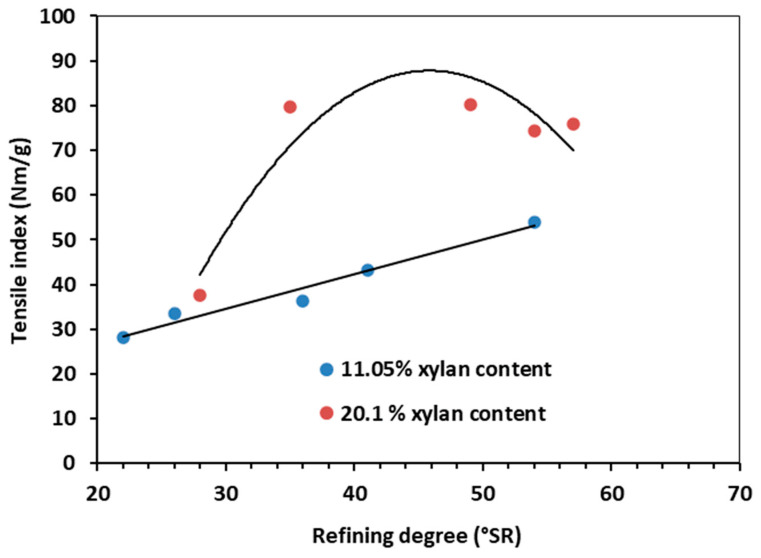
Tensile index vs. refining degree. Influence of HC (xylan) content.

**Figure 6:**
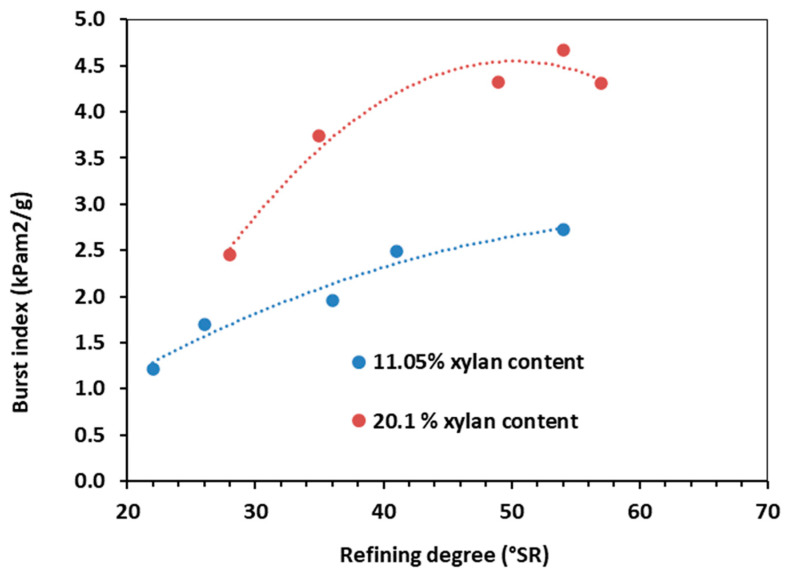
Burst index vs. refining degree. Influence of HC (xylan) content.

After obtaining the chemical composition, the obtained pulp was refined. Refining is recognized as a key unit process in the paper industry. It has a set of steps that are designed to realize the best papermaking properties: (i) fiber swelling; (ii) fibrillation (internal and external); (iii) formation of fines; and (iv) shortening of fibers [79,80,81,82,83]. Being one of the most energy-intensive processes, improved refinability means achieving a specific, optimal refining degree as fast as possible. Since xylan is the major HC constituent, the refining degree, the TI, and BI are further reported (Figure 4, Figure 5 and Figure 6) as a function of xylan composition.

Figure 4 displays the evolution of refining degree as a function of beating intensity expressed as a total number of revolutions. It can be observed that the HAEopt pulp sample has a higher initial degree of refining, even in the unbeaten state. This may be explained by the increased swelling effect of acidic hemicellulosic groups [83].

The contribution of HC in enhanced fiber–fiber bonding and the diminished fiber swelling effect on HCE pulp samples may be responsible for the differences among the reported mechanical characteristics for the analyzed pulp samples in the unbeaten state [80]. Owing to its superior xylan content, the HAE^opt^ pulp showed a faster increase in refining degree than HCE pulp, supporting the existing literature [81]. 

The mechanical characteristics under investigation (TI, BI) exhibited similar evolutions in relation to refining degree (Figure 5 and Figure 6). For HCE pulp (low xylan content), the TI-refining degree relationship is practically linear, whereas, for HCE^opt^, the dependence has a maximum point around 45 °SR (Figure 5). At this peak value, the TI of HAE^opt^ pulp is almost double in comparison with the HCE pulp. The BI showed a similar evolution. The BI-refining degree dependence is almost linear (Figure 6) for the HCE pulp, with a maximum value of ~50 °SR for the pulp with higher xylan content (HAE^opt^). 

The slight decrease in TI and BI for the HAE^opt^ pulp after reaching maximum values at ~45, ~50 °SR could be attributed to the shortening of fibers through cutting effects in the final refining stages [82]. Several explanations for the behavior of the investigated pulps also center on the combined impacts of HC concentration and refinement [79,80,81,82,83]. 

### 3.6. The Influence of Severity Factor on HC Chemical Composition and Recovery Yields 

The SF quantifies the combined effect of time and temperature (Equation (5)), and it was introduced to compare pretreatment yields conducted using different conditions [84,85]. Such comparison is presented in Table 10, using samples recovered after various extraction procedures presented in this study. It is important to note that small variations in the SF value (from 2.12 to 2.65) might result in large variations in the HCRY (from 77.5% to 69.43%) for the same extraction process. 

The highest HCRY% value was obtained at the lowest SF, for the hot alkaline treatment from the extraction liquor. At the same value of SF (2.12), the total amount of extracted polysaccharides increased significantly (from 61.63% to 74.66%) when the NaOH concentration was increased from 0.9% to 5% (HAE vs. HCE). This aligns with earlier results made during the optimization phase, which indicated that the NaOH concentration is the process parameter with the most significant impact. 

### 3.7. Characterization of HC Samples by FTIR and 1H-NMR Spectroscopy

**Figure 7:**
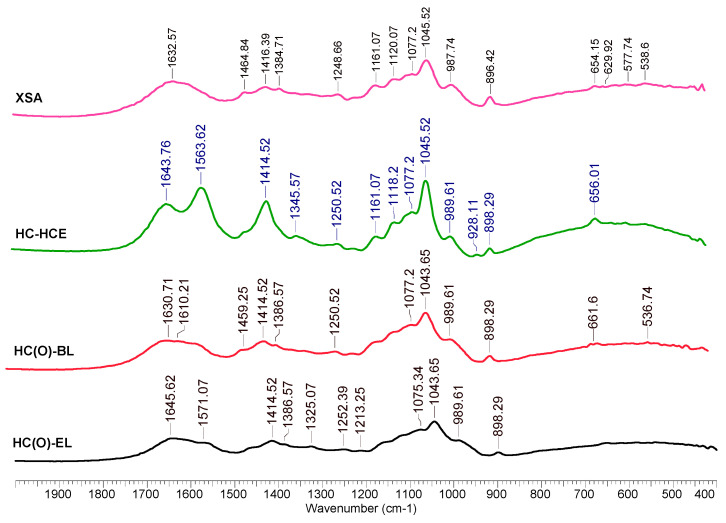
FTIR spectra of separated HC.

All of the analyzed infrared spectra of the HC presented bands occurring at ~3400 cm^−1^ (Figure 7) that were assigned to the stretching vibrations of the O-H groups and also the band occurring at ~2950 cm^−1^ that is generally assigned to the -CH_2_ antisymmetric stretching, while the band at 2850 cm^−1^ was a result of -CH_2_ symmetric stretching—this portion of the spectra is not shown in the figure for a better view of the range 1600–400 cm^−1^. All of the HC samples presented bands specific to polysaccharides: the band occurring at ~1630 cm−1 was assigned to the absorbed water [86]; The bands occurring ~1560 and ~1414 were assigned to glucuronic acid carboxylates [87]; the minor band occurring at about 1450 cm^−1^ in some samples could be assigned to the presence of the methyl groups; spectral peaks that are visible at ~1070 and ~1045 cm^−1^ of C-O stretching in the C-O-C ether linkages (the first is the inter sugar units and the second results from intra sugar (in alcoholic functional group). The peaks at ~898 cm^−1^ were attributed to the stretching vibration modes (both symmetric and antisymmetric) of C-O in the ether linkage and are considered specific to the β-1-4 bonds between xylose units of the xylan chain [88]. Other bands at lower wavenumbers, such as ~690 cm^−1^, are attributed to the out-of-plane C-H deformations.

**Figure 8:**
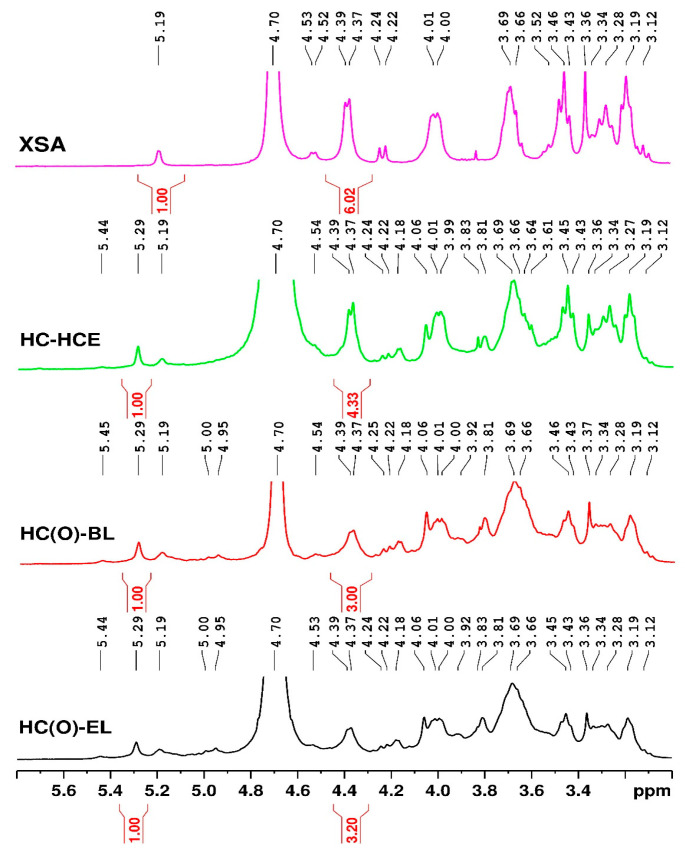
^1^H-NMR spectra of the separated hemicelluloses.

NMR spectroscopy was used to study the molecular structure of hemicelluloses. Figure 8 presents the proton NMR spectra of three samples of extracted HC and a commercial xylan sample (X4252 10G). The spectral pattern typical for the proton in HC has signals in the chemical shift region between 3.12–5.45 ppm due to xylose, arabinose, and glucuronic acid residues [89]. The main spectral characteristics of the analyzed HC samples and commercial xylan are shown in Table 11.

The protons from β-(1→4)-D-xylopyranose (β-D-Xylp or (X)) units have signals at 4.39 (H1), 4.01 (H5eq), 3.69 (H4), 3.45 (H3), 3.28 (H5ax) and 3.19 ppm (H2), being found in a significant amount. As shown in Figure 8, the HC samples have more signals in the range 4.9–5.5 ppm compared with the XSA spectrum. Thus, the signals at 5.44 (H1) and 3.34 (H3) ppm are due to the 2-α-L-arabinofuranosyl units (2-α-Araf) and the terminating xylopyranose units (Xylp) from β-Xylp-(1→2)-α-Araf-(1→3), respectively. 

The sharp peaks at 5.29 ppm (H1), together with signals at 4.18 (H4), 4.06 (H2) and 3.83 ppm (H3) are attributed to α-L-arabinofuranosyl(1→3)-linkage with the mono-substituted β-D-Xylopyranose unit in the main chain (4-O-methyl glucuronic acid residue substituted xylose residues or (XG) [89], being in a significant proportion. 

Peaks from 4.95–5.0 ppm and 4.53 ppm are attributed to H3 and H1 protons of 2-O-acetylated internal xylose residues (Xylp-3Ac). The signal at 5.19 ppm is assigned to the anomeric proton from 4-O-methyl-α-D-glucuronic acid (4-O-Me-α-D-GlcpA or (G)), together with the resonances at 4.2 (H5), 3.66 (H3), 3.43 (H2), 3.36 (-OCH_3_) and 3.12 ppm (H4), all these signals being observed also in XSA spectrum. The ratio of xylose units (X) and 4-O-methyl glucuronic acid (G) was determined using the integration of the corresponding anomeric protons [90,91,92]. 

**Table 11:** Synthetic presentation of the 1H-NMR peak assignments.

Spectral Range/Chemical Shift	Assignment in Samples	Literature Data
non substituted xylose residues (X)	−4.39 ppm H1 −3.19 ppm H2 −3.28 ppm H5 axial −3.45 ppm H3 −3.69 ppm H4 −4.01 ppm H5 eq	4.4 [89] 3.21 3.3 3.48 3.71 4.03
4-O-methyl glucuronic acid residue substituted xylose residues (XG)	−5.29 ppm H1 −4.06 ppm H2 −3.83 ppm H3 −4.18 ppm H4	5.31 [89] 4.08 3.83 4.2
4-O-methyl-glucuronic acid residue (G)	−3.36 ppm –OCH3 −3.12 ppm H4 −3.43 ppm H2 −5.19 ppm H1 −3.66 ppm H3 −4.2 ppm H5	3.34 [93] 2.95 [94] 3.43 [95] 5.21 [89] 3.64 [95] 4.34/4.37 [95]

The different values of integrals for the range 4.2–4.4 ppm specific for the H1 XG signals reside in the HC extraction condition or source. In the case of the HC(O)-EL sample, a value of 3.2 was obtained. A smaller value is observable for the HC(O)-BL sample. This suggests a decrease in the number of 4-O-methyl glucuronic acid residue substituted anhydro-xylose residues as a result of the increase in the temperature (from 100 °C to 140 °C) in the reaction environment and intensification of the ether linkage cleavage in the alkaline pulping environment. The increase in C_NaOH_ from 1% to 5% causes liberation of different structure HC—this is why integral values for the H1 XG signals are increased in the sample denoted HC-HCE. The value of 6.0 of this integral obtained for the spectrum of beechwood xylan XSA suggests an even higher presence of XG groups in this sample, a feature which is common for hardwood hemicelluloses [96].

### 3.8. Comparison with Similar Studies 

Several literature studies report similar attempts to produce both HC and/or HC derivatives and paper pulp with satisfactory mechanical properties starting from agro-wastes. Some focus on CS as raw material, but the extraction procedures differ from the current study. However, the results are promising regardless of the pretreatment and extraction procedure used. The CS are an attractive raw material for the simultaneous production of HC and/or HC derivatives and paper pulp (Table 12). 

Our study’s findings corroborate those of other authors who have noted a decline in paper quality in response to increasing extraction HC yields. Hot water extraction was utilized by Chen and colleagues [101] to extract sugars from CS, yielding a yield of xylose extraction of 70.2%; however, various paper quality indices, including brightness, BI, and breaking length, were negatively affected. 

## 4. Conclusions

This work aimed to identify the best-suited strategy for the complete valorization of CS and focused on the optimum equilibrium between the amount of extracted HC and paper quality. To reach this objective, two different stages were applied (screening and optimization).

The screening phase revealed that conventional HAE at high alkali concentrations, which yields better results in terms of HC extraction but suffers from lowered pulp yield and papermaking properties, should not be considered as part of the pathway for the co-production of HC and papermaking pulp by using CS as raw material. The significant reduction in paper quality and DP directly results from the high C_NaOH_. Therefore, lower alkali concentrations are recommended. The HWT treatment, although performed in relatively low severity, also inflicts significant mechanical strength losses. In this aspect, the preliminary HAE hemicellulose extraction using low alkali concentrations of liquors similar to those used in soda pulping situations seemed to show the promised result.

The RSM strategy showed the influence of the chosen factors: temperature, C_NaOH,_ and time on the modeled system responses. Depending on the targeted yields’ values, the model equation could be used to predict the system output under different circumstances. The resulting models were experimentally validated. A more aggressive extraction procedure (HCE) was used for comparison purposes, showing that a high HC extraction yield is detrimental to paper properties. The observed differences in pulp refinability and papermaking properties directly result from HC participation in refining stages and in developing sheet strength.

## Figures and Tables

**Table 1:** Independent variables and their variation range for HAE_low_.

Independent Variables	Units		Range		Symbol
			From	To	
Temperature	(°C)	X1	100	130	T
NaOH concentration	% wt.	X2	0.6	1.2	C_NaOH_
Duration (time) of HAE	minutes	X3	30	90	t

**Table 2:** Chemical composition (% wt.) of corn stalks and HWT solid residue.

Material	Glucan	Xylan	Arabinan	PStot	AIL	ASL	AE	Ash	Ref.
CS	39.71	19.82	4.28	63.81	20.62	1.60	4.36	7.05	This work
HWT CS	47.68	16.44	5.43	69.55	23.59	1.37	n.d.	1.50	
CS	36.80	19.90	3.20	59.90	20.10		11.80	1.00	[75]
CS	34.40	15.88	n.d.	-	14.10	2.48	11.84	3.04	[76]
CS	36.80	21.70	2.60	61.10	17.20		n.d.	n.d.	[77]
CS	42.10	22.90	2.90	67.90	17.50	-	9.80	4.20	[78]

n.d. = not determined.

**Table 3:** Chemical composition (% wt.) of the obtained solid material—papermaking fibers (pulp).

Treatment Param.	Glucan	Xylan	Arabinan	SY (%)	AIL	ASL
HAE^high^, 5%NaOH	63.58	12.75	5.67	33.60	6.80	0.75
HAE^high^, 3%NaOH	64.50	13.90	6.55	35.50	8.80	0.95
SP, raw CS	68.70	18.9	2.74	48,30	9.95	1.05
HAE_low_–SP	67.50	16.47	3.30	47.13	10.71	1.12
HWT–SP	66.04	19.40	2.72	46.65	11.99	1.22

**Table 4:** Polymerization degree and paper properties as a function of treatment sequence.

Treatment Param.	DP	TI, (N·m/g)	BI, (kPa·m^2^/g)
HAEhigh, 5%NaOH	750	72.40	2.56
HAEhigh, 3%NaOH	880	62.90	3.62
SP, raw CS	1047	83.20	4.71
HAElow–SP	925	75.50	4.35
HWT–SP	830	70.40	3.11

**Table 5:** Chemical composition ((% wt.) of the hemicelluloses isolated from HAE extraction liquors and subsequent pulping black liquor.

Treatment Parameters	Glucan	Xylan	Arabinan	DP
HAE^high^, 5%NaOH, EL	3.11	65.14	5.24	290
HAE^high^, 3%NaOH, EL	2.85	62.47	8.95	285
SP, raw CS, BL	4.34	54.40	14.10	273
HAE^low^, EL	4.80	49.06	17.93	320
HAE_low_–SP, BL	5.70	61.82	14.51	262
HWT, EL	11.27	7.70	1.28	n.d.
HWT–SP, BL	3.35	57.20	12.24	230
X4252 10G	0.05	91.34	2.09	255

**Table 6:** Experimental planning and experimental results.

Exp No.	T, (°C)	C_NaOH_, (%)	T, (min)	XHC, (%)	XRCS, (%)	TI, (N·m/g)	BI, (kPa·m^2^/g)
1	100	0.6	30	44.74	19.22	70.90	2.62
2	100	0.6	60	54.53	22.91	75.50	3.46
3	100	0.6	90	54.53	25.62	74.30	3.49
4	115	0.6	60	41.29	21.60	68.92	4.24
5	130	0.6	30	41.16	27.43	63.80	4.56
6	130	0.6	90	45.26	29.25	74.29	4.85
7	100	0.9	60	52.41	25.32	75.61	4.48
8	115	0.9	60	51.62	26.91	67.90	4.25
9	115	0.9	30	50.96	26.68	67.30	3.82
10	115	0.9	60	42.88	27.31	67.20	4.35
11	115	0.9	60	62.74	27.53	68.11	4.29
12	115	0.9	60	57.57	27.21	69.10	4.32
13	115	0.9	60	54.40	29.02	68.20	4.10
14	115	0.9	60	52.28	26.34	70.20	4.45
15	115	0.9	60	52.15	25.93	66.91	4.25
16	115	0.9	90	53.21	27.62	64.52	4.19
17	130	0.9	60	48.04	34.80	71.89	4.27
18	100	1.2	30	53.34	18.40	76.60	4.53
19	100	1.2	90	53.47	22.80	71.70	4.02
20	115	1.2	60	57.71	28.10	75.54	4.10
21	130	1.2	30	63.53	35.40	62.20	3.27
22	130	1.2	90	55.06	35.20	71.40	2.73

**Table 7:** Model equation coefficients (actual factors) and statistical parameters (Model *p* value < 0.05).

Y_i_	β_0_	X_1_	X_2_	X_3_	X_1_X_2_	X_2_X_3_	X_1_X_3_	X_1_^2^	X_2_^2^	R^2^	R^2^_adj_
Y_1_	78.2	−0.511	−54.3	0.760	0.770	−0.00396	−0.309	-	-	0.918	0.87
Y_2_	2.12	0.0306	−24.9	0.400	0.510	−0.0262	−0.0583	-	−13.8	0.915	0.872
Y_3_	221	−4.31	245	3.59	−2.62	−0.0284	−4.85	0.0268	50.7	0.95	0.899
Y_4_	−53.3	0.368	84.3	0.747	−0.729	−0.0064	−0.947	0.000847	−0.13	0.93	0.84

**Table 8:** Model validation results.

System Responses	XHC (%)	XRCS (%)	TI (N·m/g)	BI (kPa·m^2^/g)
Predicted values	51.67	21.77	78.50	4.54
Experimental values	54.64	20.54	80.10	4.33

**Table 9:** Chemical composition of HAE^opt^ and HCE pulps.

Pulp Type	SY (%)	Glucan (%)	Xylan (%)	Arabinan (%)	AIL	ASL
HAE^opt^	42.74	67.10	20.10	2.60	6.50	1.10
HCE	64.10	80.41	11.05	1.40	4.30	0.70

**Table 10:** Hemicellulose recovery yields and chemical composition (% wt.) as a function of severity factor.

Sample Source	SF	HCRY (%)	Glucan	Xylan	Arabinan	Pstot (%)
HAE, 0.9% NaOH, EL	2.12	77.50	3.23	54.28	4.12	61.63
	2.54	76.92	3.83	53.96	3.64	59.75
	2.65	69.43	3.47	52.29	3.45	59.21
HAE, 0.9% NaOH, BL	3.39	73.09	4.01	63.26	5.49	72.76
	3.45	72.82	3.69	68.97	5.60	78.25
	3.55	65.92	5.94	61.80	6.09	83.82
HAE^opt^, EL	2.02	79.23	4.12	54.64	5.03	63.79
HAE^opt^, BL	3.31	67.48	3.02	67.84	4.85	75.71
HCE, 5%NaOH, EL	2.12	72.34	7.22	65.07	2.37	74.66

**Table 12:** Comparison with similar studies *.

Raw Material	Extraction/Pretreatment	HC Derivatives	TI (N·M/G)	BI (kPa·m^2^/g)	Ref.
CS	HAE	54.64% xylan	80.10	4.33	This work
CS	One-step formic acid	61.00% xylose	50.10	3.00	[49]
CS and kash	Soda-anthraquinone pulping	-	56.00	4.70	[97]
CS	Alkaline sulfite pulping	-	62.40	3.80	[98]
CS	Alkaline sulfite cooking	118.40 g xylose/kg CS	97.40	5.20	[41]
Corn cobs	Hydrothermal pretreatment	52.35% furfural	43.00	-	[99]
CS	Acid pre-impregnated steam explosion	acetone: 0.09 g/g, butanol: 0.18 g/g, ethanol: 0.04 g/g	24.00	0.99	[100]

* best results reported.

## Data Availability

Data will be available upon request.

## Supplementary Materials

The following supporting information can be downloaded at: https://www.mdpi.com/article/10.3390/polym15234597/s1,: Figure S1: Corn stalks chopped and shredded; Figure S2: The pulping reactor; Figure S3: Various stages of liquid phase processing; Figure S4: Hemicelluloses samples (before and after drying); Figure S5: Paper sheets and preliminary strength testing; Supplementary File S1: ANOVA; Supplementary File S2: 1H-NMR spectra; Supplementary File S3: FTIR data.
