# Supplementary material for: Integrated Hemicellulose Extraction and Papermaking Fiber Production from Agro-Waste Biomass
Source: Polymers (Basel). 2023 Dec 1;15(23):4597. doi: 10.3390/polym15234597 (PMC10708159; doi:10.3390/polym15234597)
Supplement: Supplementary file 1 [file polymers-15-04597-s001.zip › polymers-2711413-supplementary revised.pdf]

Supplementary Materials: Integrated hemicellulose extraction and papermaking fiber production from agro-waste biomass

Adrian Cătălin Puițel, Cătălin Dumitrel Balan, Gabriela-Liliana Ailiesei, Elena Niculina Drăgoi, Mircea Teodor Nechita

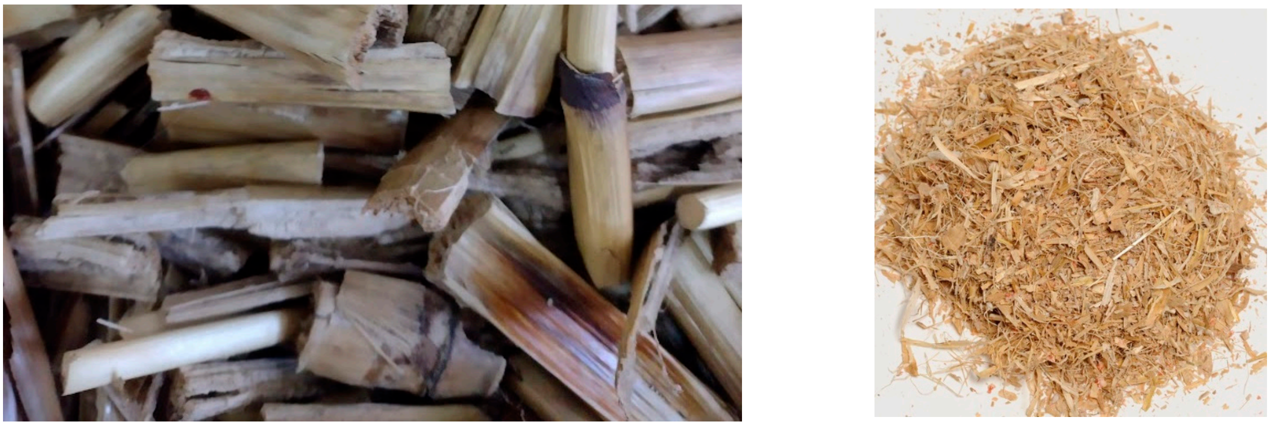

Figure S1. Corn stalks chopped and shredded.

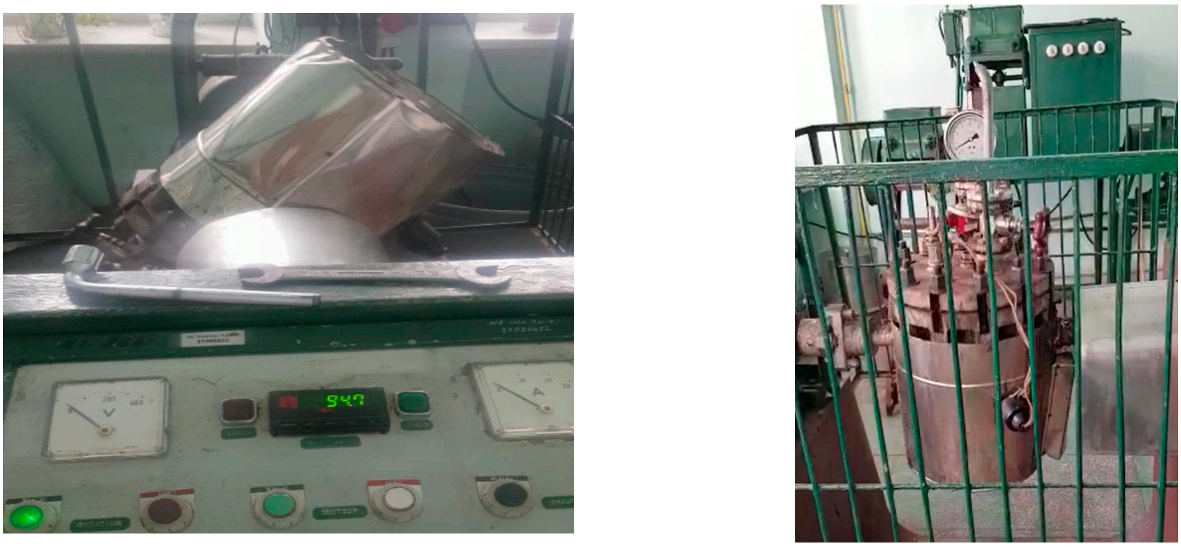

Figure S2. The pulping reactor.

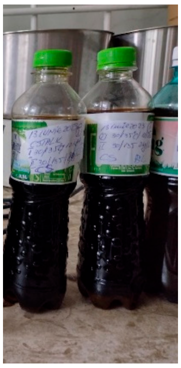
Storage bottles

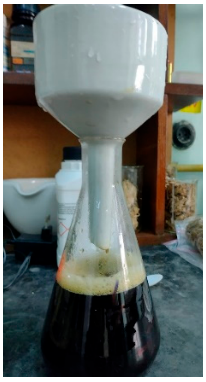
Fines removal (filtering)

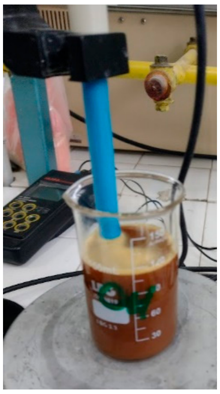
Lignin precipitation

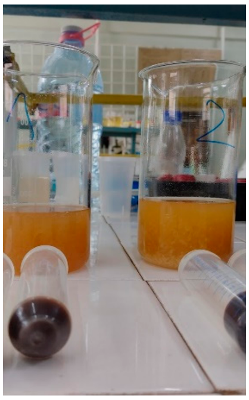
Hemicellulose separation

Figure S3. Various stages of liquid phase processing.

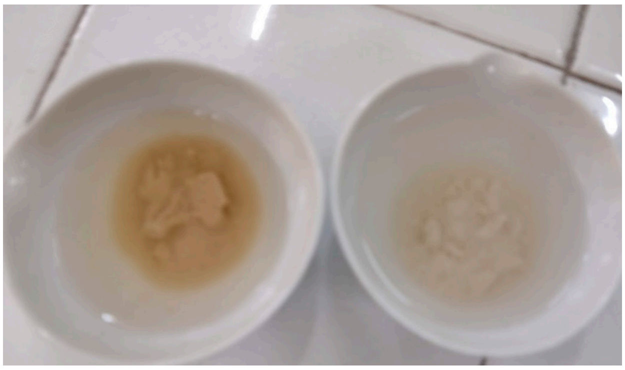
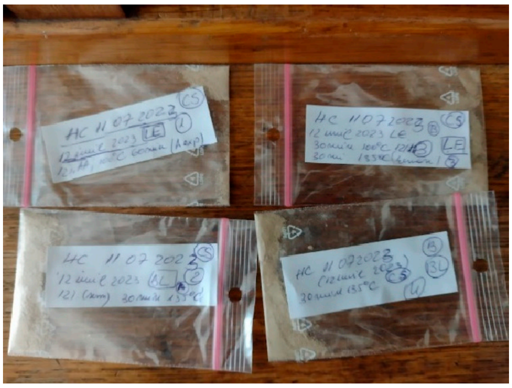

Figure S4. Hemicelluloses samples (before and after drying).

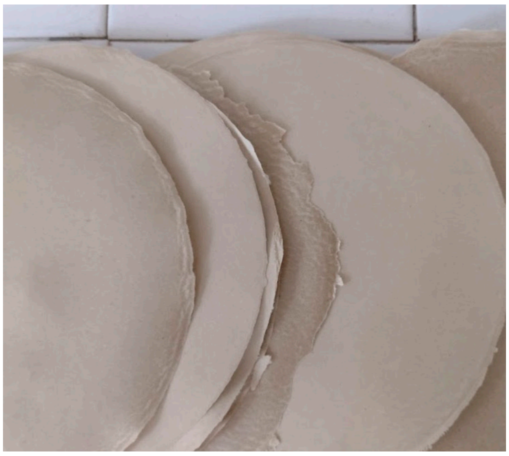
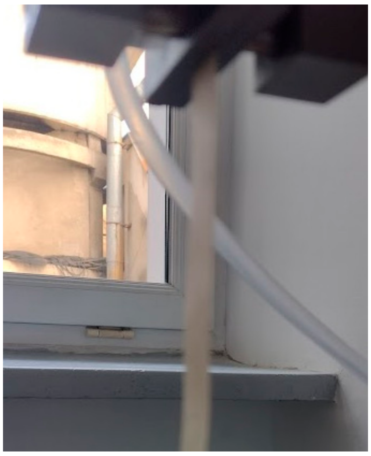

Figure S5. Paper sheets and preliminary strength testing.
