# Supplementary material for: Integrated Hemicellulose Extraction and Papermaking Fiber Production from Agro-Waste Biomass
Source: Polymers (Basel). 2023 Dec 1;15(23):4597. doi: 10.3390/polym15234597 (PMC10708159; doi:10.3390/polym15234597)
Supplement: Supplementary file 1 [file polymers-15-04597-s001.zip › Supplementary File S1 ANOVA.pdf]

Integrated hemicellulose extraction and papermaking fiber production from agro-waste biomass

Adrian Cătălin Puițel[1], Cătălin Dumitrel Balan [1], Gabriela-Liliana Ailiesei [2], Elena Niculina Drăgoi[1], Mircea Teodor Nechita[1],

[1] “"Gheorghe Asachi” " Technical University, Iasi, Faculty of Chemical Engineering and Environmental Protection “"Cristofor Simionescu”, ", Bd. Prof. Dimitrie Mangeron, No. 73, 700050, Iaşi, România

[2] "Petru Poni" Institute of Macromolecular Chemistry, 41A Grigore Ghica Voda Alley, 700487, Iaşi, România,

Table S1. ANOVA for Response Surface 2FI Model (XHC%)

Analysis of variance table [Partial sum of squares - Type III]						
	Sum of		Mean	F	p-value	
Source	Squares	df	Square	Value	Prob > F	
Model	395.8821	6	65.98035	20.44098*	< 0.0001	significant
A-Temperature (oC)	7.520282	1	7.520282	2.329814	0.155	
B-CNaOH (%)	217.8757	1	217.8757	67.49878	< 0.0001	
C-Time (minutes)	6.24377	1	6.24377	1.934345	0.192	
AB	105.3127	1	105.3127	32.62629	0.000136	
AC	25.45098	1	25.45098	7.884816	0.0170	
BC	61.77161	1	61.77161	19.1371	0.00111	
Residual	35.50631	11	3.227847			
Lack of Fit	32.31505	9	3.590561	2.250242	0.345	not significant
Pure Error	3.191267	2	1.595633			
Cor Total	431.3884	17				

*The Model F-value of 20.44 implies that the model is significant. There is only a 0.01% chance that a "Model F-value" this large could occur due to noise.

Table S2 ANOVA analysis for XRCS%

Analysis of variance table [Partial sum of squares - Type III]						
	Sum of		Mean	F	p-value	
Source	Squares	df	Square	Value	Prob > F	
Model	381.7653	7	54.5379	21.4931*	< 0.0001	significant
A-Temperature (oC)	267.1714	1	267.1714	105.2908	< 0.0001	
B-CNaOH (%)	27.90389	1	27.90389	10.99678	0.00510	
C-Time (minutes)	18.67925	1	18.67925	7.361392	0.0168	
AB	46.22989	1	46.22989	18.21896	0.000780	
AC	11.15897	1	11.15897	4.397689	0.0546	
BC	2.205	1	2.205	0.868979	0.367	
B^2	8.435116	1	8.435116	3.324234	0.0897	
Residual	35.52446	14	2.537461			
Lack of Fit	29.59366	9	3.288184	2.772125	0.137	not significant
Pure Error	5.9308	5	1.18616			
Cor Total	417.2898	21				

* The Model F-value of 21.49 implies that the model is significant.

Table S3 ANOVA for Response Surface Reduced Cubic Model (tensile index)

Analysis of variance table [Partial sum of squares - Type III]						
	Sum of		Mean	F	p-value	
Source	Squares	df	Square	Value	Prob > F	
Model	300.0538328	9	33.33931	18.83502*	< 0.0001	significant
A-Temperature (oC)	6.890006722	1	6.890007	3.892503	0.0800	
B-CNaOH (%)	23.64987847	1	23.64988	13.36098	0.00527	
C-Time (minutes)	7.751168181	1	7.751168	4.379016	0.0659	
AB	1.06579121	1	1.065791	0.602118	0.458	
AC	8.463906209	1	8.463906	4.781676	0.0566	
BC	15.06108267	1	15.06108	8.508746	0.0171	
A^2	90.36291695	1	90.36292	51.05045	< 0.0001	
B^2	51.81776624	1	51.81777	29.2744	0.000427	
Residual	15.93063781	9	1.770071			
Lack of Fit	6.872499529	2	3.43625	2.655485	0.139	not significant
Pure Error	9.058138282	7	1.29402			
Cor Total	315.9844706	18				

*The Model F-value of 18.84 implies that the model is significant

Table S4 ANOVA for Response Surface Reduced Cubic Model (burst index)

Analysis of variance table [Partial sum of squares - Type III]						
	Sum of		Mean	F	p-value	
Source	Squares	df	Square	Value	Prob > F	
Model	3.861846723	9	0.429094	15.36422*	0.000191	significant
A-Temperature (oC)	0.021907626	1	0.021908	0.784428	0.399	
B-CNaOH (%)	0.003275195	1	0.003275	0.117272	0.740	
C-Time (minutes)	0.028211495	1	0.028211	1.010146	0.341	
AB	0.678003123	1	0.678003	24.2767	0.000817	
AC	0.18975851	1	0.189759	6.794527	0.0284	
BC	0.003268068	1	0.003268	0.117017	0.740	
A^2	0.09029045	1	0.09029	3.232956	0.106	
B^2	0.000339045	1	0.000339	0.01214	0.915	
Residual	0.251353277	9	0.027928			
Lack of Fit	0.179569944	2	0.089785	8.755442	0.0124	significant
Pure Error	0.071783333	7	0.010255			
Cor Total	4.1132	18				

* The Model F-value of 15.36 implies that the model is significant
