# Supplementary material for: Integrated Hemicellulose Extraction and Papermaking Fiber Production from Agro-Waste Biomass
Source: Polymers (Basel). 2023 Dec 1;15(23):4597. doi: 10.3390/polym15234597 (PMC10708159; doi:10.3390/polym15234597)
Supplement: Supplementary file 1 [file polymers-15-04597-s001.zip › Supplementary File S2 1H-NMR spectra.pdf]

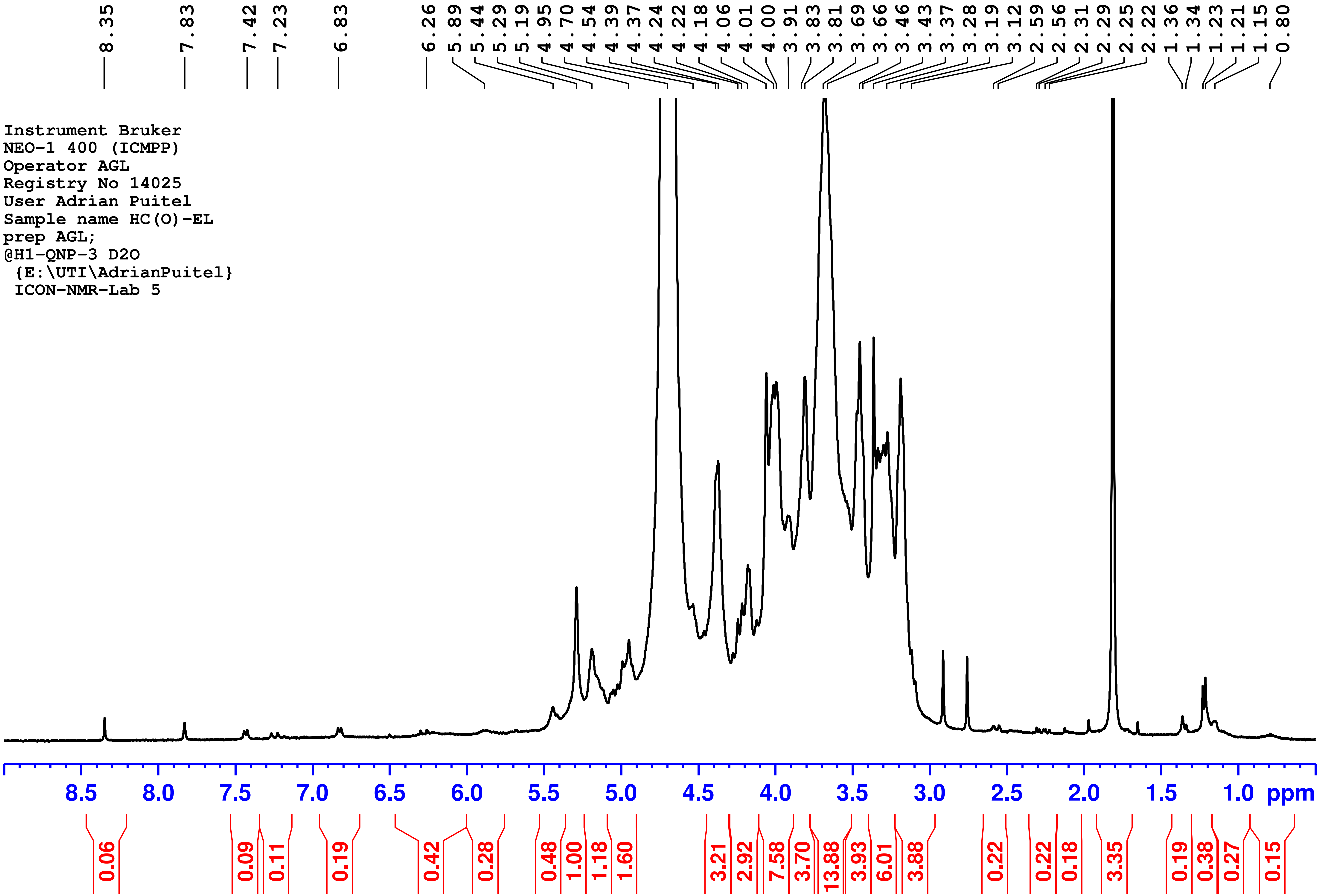
Instrument Bruker
NEO-1 400 (ICMPP)
Operator AGL
Registry No 14025
User Adrian Puitel
Sample name HC(O)-EL
prep AGL;
@H1-QNP-3 D2O
{E:\UTI\AdrianPuitel}
ICON-NMR-Lab 5
8.35 7.83 7.42 7.23 6.83 6.26 5.89 5.44 5.29 5.19 4.95 4.70 4.54 4.39 4.37 4.24 4.22 4.18 4.06 4.01 4.00 3.91 3.83 3.81 3.69 3.66 3.46 3.43 3.37 3.28 3.19 3.12 2.59 2.56 2.31 2.29 2.25 2.22 1.36 1.34 1.23 1.21 1.15 0.80
8.5 8.0 7.5 7.0 6.5 6.0 5.5 5.0 4.5 4.0 3.5 3.0 2.5 2.0 1.5 1.0 ppm
0.06 0.09 0.11 0.19 0.42 0.28 0.48 1.00 1.18 1.60 3.21 2.92 7.58 3.70 13.88 3.93 6.01 3.88 0.22 0.22 0.18 3.35 0.19 0.38 0.27 0.15

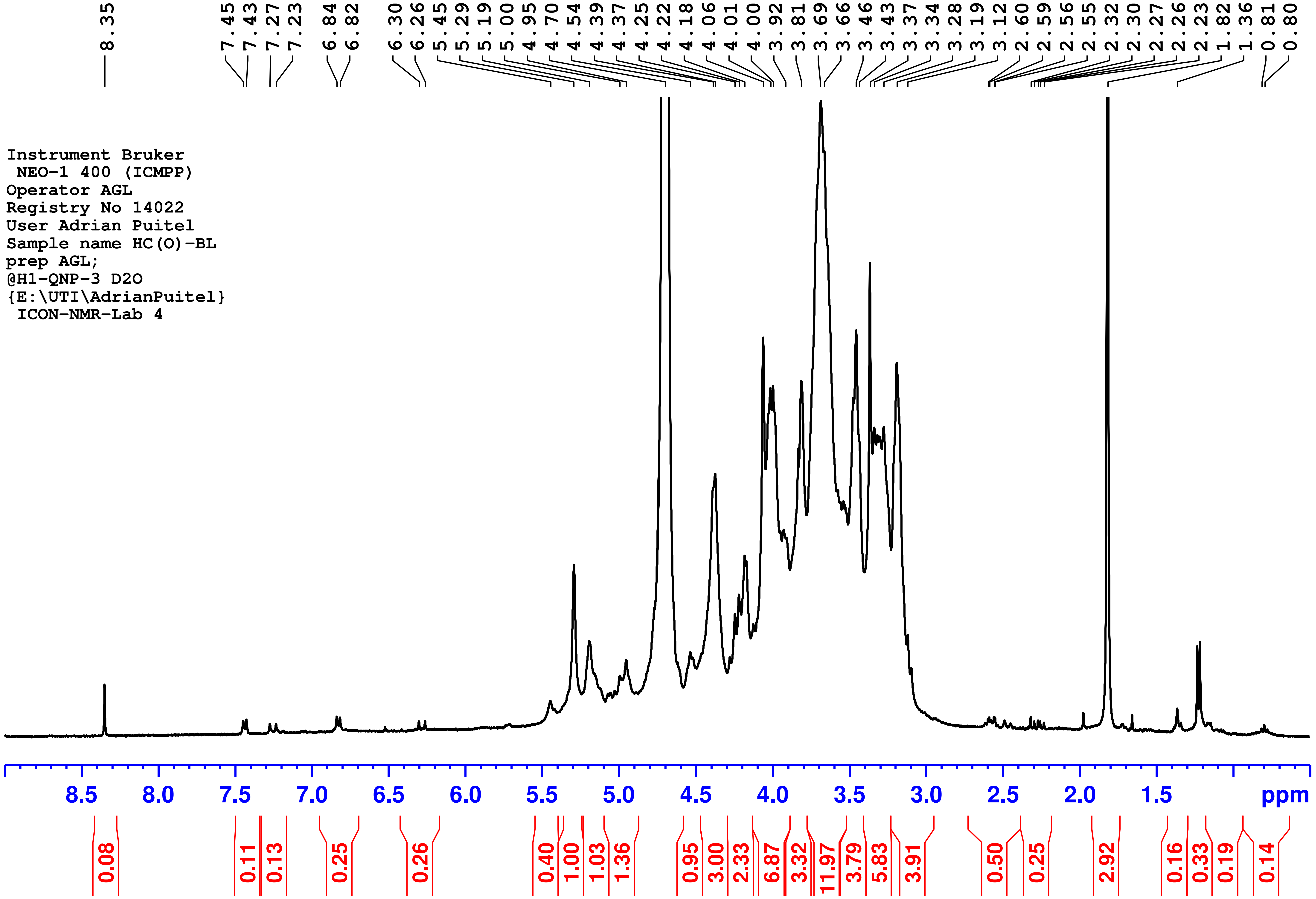
8.35
7.45 7.43 7.27 7.23 6.84 6.82 6.30 6.26 5.45 5.29 5.19 5.00 4.95 4.70 4.54 4.39 4.37 4.25 4.22 4.18 4.06 4.01 4.00 3.92 3.81 3.69 3.66 3.46 3.43 3.37 3.34 3.28 3.19 3.12 2.60 2.59 2.56 2.55 2.32 2.30 2.27 2.26 2.23 1.82 1.36 0.81 0.80
Instrument Bruker
NEO-1 400 (ICMPP)
Operator AGL
Registry No 14022
User Adrian Puitel
Sample name HC(O)-BL
prep AGL;
@H1-QNP-3 D2O
{E:\UTI\AdrianPuitel}
ICON-NMR-Lab 4
8.5 8.0 7.5 7.0 6.5 6.0 5.5 5.0 4.5 4.0 3.5 3.0 2.5 2.0 1.5 ppm
0.08 0.11 0.13 0.25 0.26 0.40 1.00 1.03 1.36 0.95 3.00 2.33 6.87 3.32 11.97 3.79 5.83 3.91 0.50 0.25 2.92 0.16 0.33 0.19 0.14

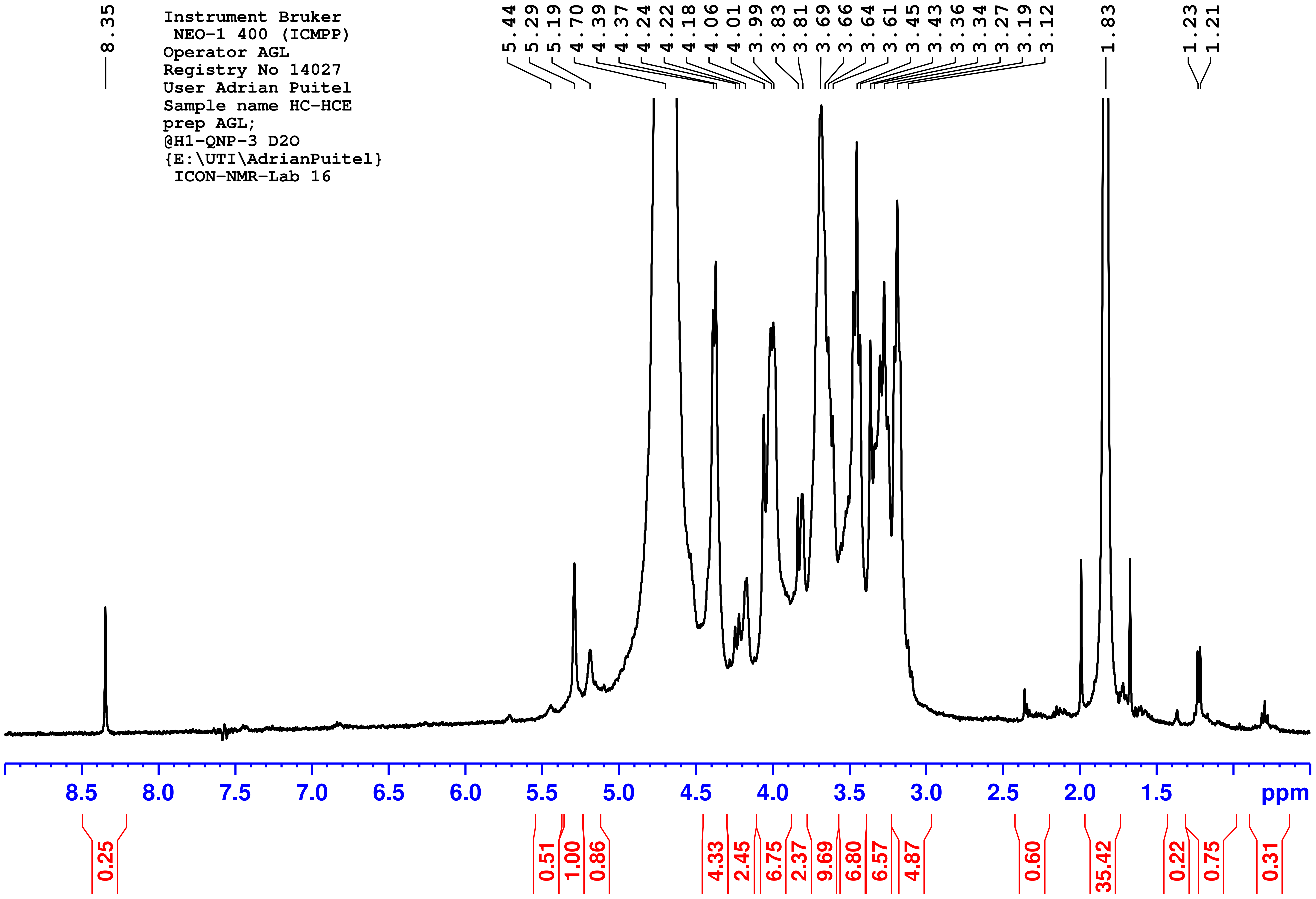
Instrument Bruker
NEO-1 400 (ICMPP)
Operator AGL
Registry No 14027
User Adrian Puitel
Sample name HC-HCE
prep AGL;
@H1-QNP-3 D2O
{E:\UTI\AdrianPuitel}
ICON-NMR-Lab 16
8.35
5.44
5.29
5.19
4.70
4.39
4.37
4.24
4.22
4.18
4.06
4.01
3.99
3.83
3.81
3.69
3.66
3.64
3.61
3.45
3.43
3.36
3.34
3.27
3.19
3.12
1.83
1.23
1.21
8.5
8.0
7.5
7.0
6.5
6.0
5.5
5.0
4.5
4.0
3.5
3.0
2.5
2.0
1.5
ppm
0.25
0.51
1.00
0.86
4.33
2.45
6.75
2.37
9.69
6.80
6.57
4.87
0.60
35.42
0.22
0.75
0.31

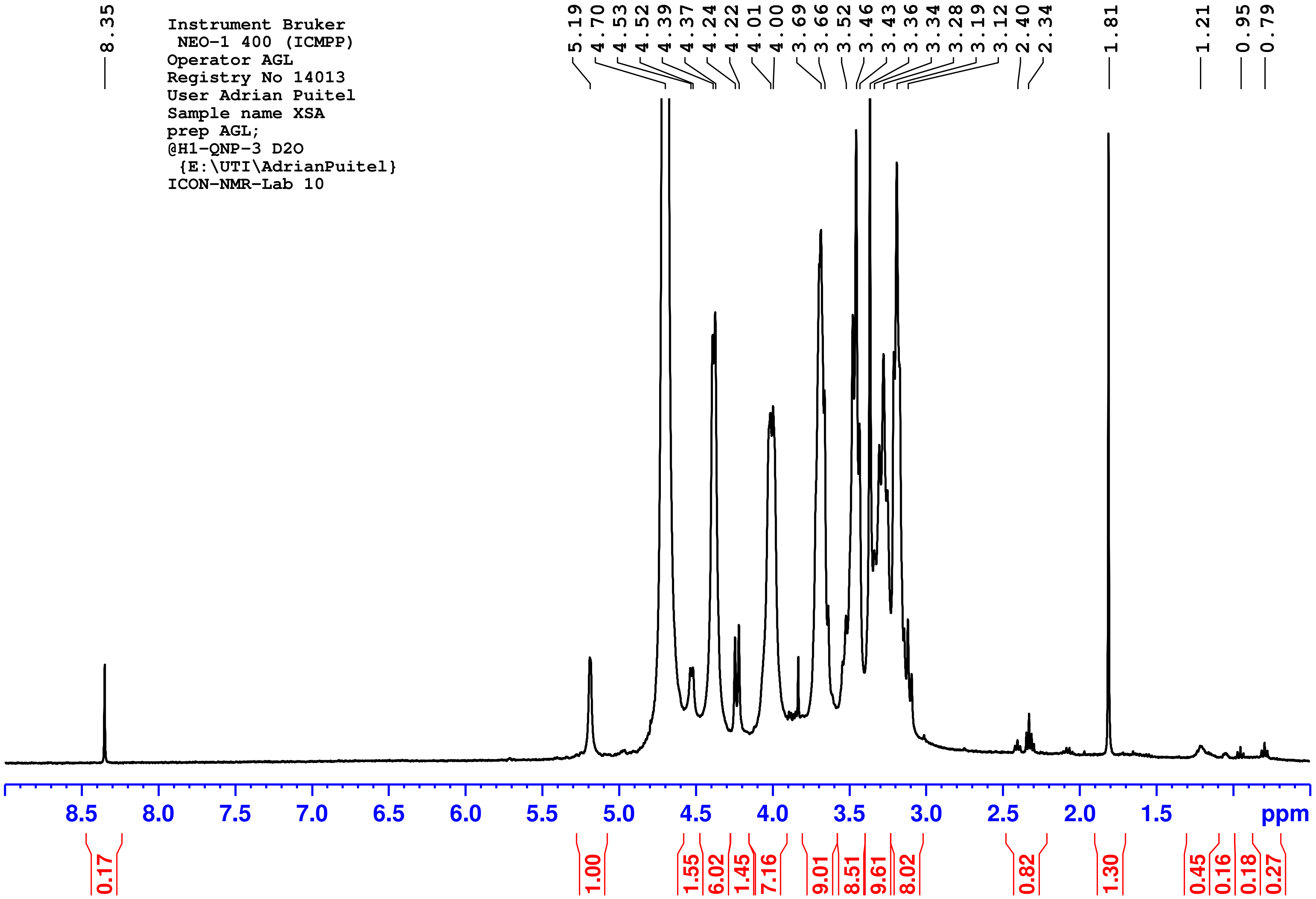
Instrument Bruker
NEO-1 400 (ICMPP)
Operator AGL
Registry No 14013
User Adrian Puitel
Sample name XSA
prep AGL;
@H1-QNP-3 D2O
{E:\UTI\AdrianPuitel}
ICON-NMR-Lab 10
8.35
5.19 4.70 4.53 4.52 4.39 4.37 4.24 4.22 4.01 4.00 3.69 3.66 3.52 3.46 3.43 3.36 3.34 3.28 3.19 3.12 2.40 2.34
1.81
1.21 0.95 0.79
8.5 8.0 7.5 7.0 6.5 6.0 5.5 5.0 4.5 4.0 3.5 3.0 2.5 2.0 1.5 ppm
0.17 1.00 1.55 6.02 1.45 7.16 9.01 8.51 9.61 8.02 0.82 1.30 0.45 0.16 0.18 0.27
