# Supplementary material for: Integrated Hemicellulose Extraction and Papermaking Fiber Production from Agro-Waste Biomass
Source: Polymers (Basel). 2023 Dec 1;15(23):4597. doi: 10.3390/polym15234597 (PMC10708159; doi:10.3390/polym15234597)

File Name	E:\SUSTECLCR PED 2023\DISEMINARE 2023\ARTICOL DATE 2023\SELECTATE PENTRU SUPLEMENTARY\FTIR DATA\HC_(O)EL_9-28-2023T9-32-39 AM.SPC		
Date	28 Sep 2023 09:33:52	***Technique***	Infrared
Spectral Region	IR	***X Axis***	Wavenumber (cm-1)
Y Axis	Absorbance	***Spectrum Range***	400.6890 - 3999.4336
Points Count	1932	***Data Spacing***	1.8637

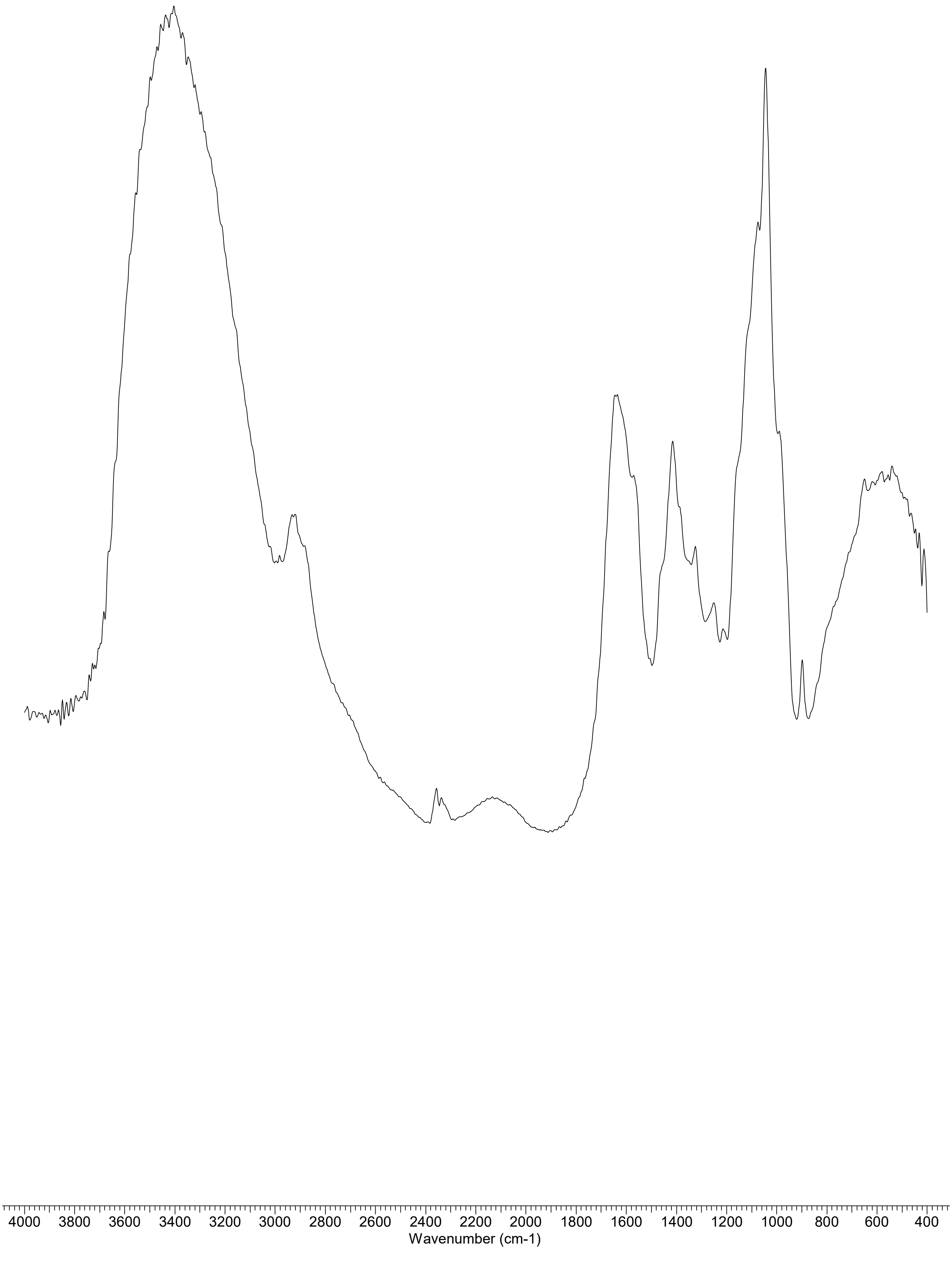

File Name	E:\SUSTECLCR PED 2023\DISEMINARE 2023\ARTICOL DATE 2023\SELECTATE PENTRU SUPLEMENTARY\FTIR DATA\HC_(O)BL_9-28-2023T9-46-20 AM.SPC		
Date	28 Sep 2023 09:46:50	Technique	Infrared
Spectral Region	IR	X Axis	Wavenumber (cm-1)
Y Axis	Absorbance	Spectrum Range	400.6890 - 3999.4336
Points Count	1932	Data Spacing	1.8637

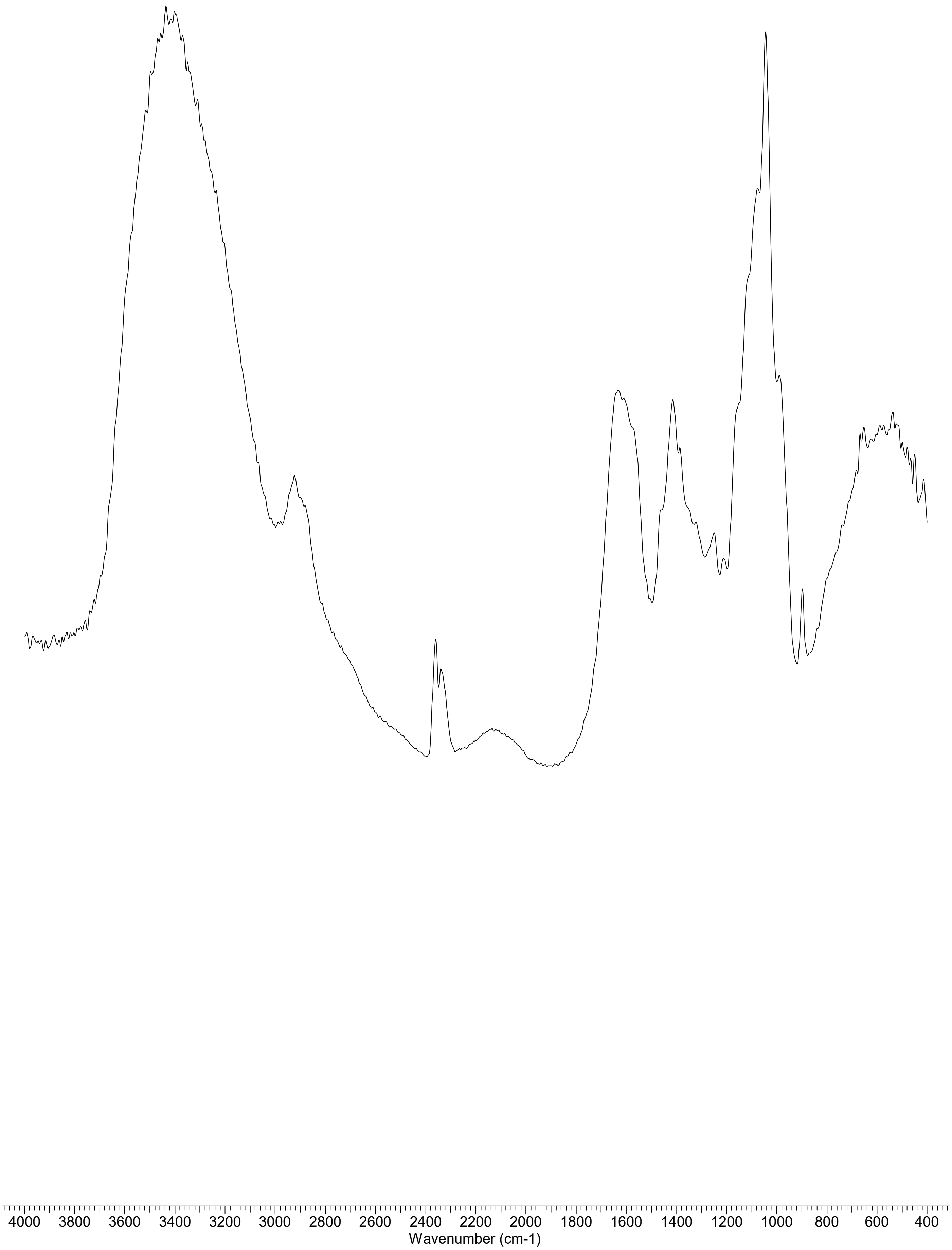

File Name	E:\SUSTECLCR PED 2023\DISEMINARE 2023\ARTICOL DATE 2023\SELECTATE PENTRU SUPLEMENTARY\FTIR DATA\HC_HCE_9-28-2023T9-24-24 AM.SPC		
Date	28 Sep 2023 09:25:46	Technique	Infrared
Spectral Region	IR	X Axis	Wavenumber (cm-1)
Y Axis	Absorbance	Spectrum Range	400.6890 - 3999.4336
Points Count	1932	Data Spacing	1.8637

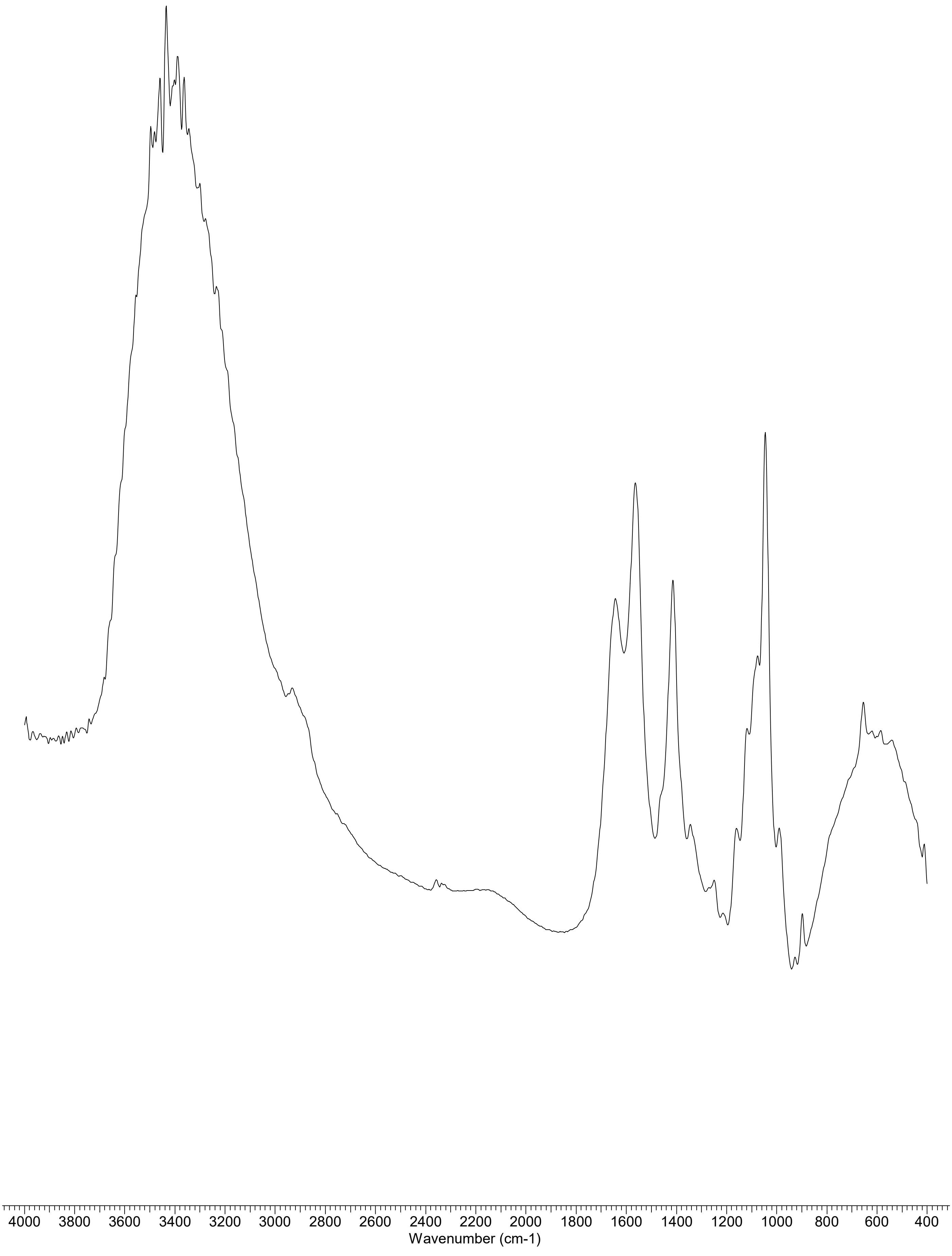

File Name	E:\SUSTECLCR PED 2023\DISEMINARE 2023\ARTICOL DATE 2023\SELECTATE PENTRU SUPLEMENTARY\FTIR DATA\XSA.SPC		
Date	02 Oct 2023 13:26:56	***Technique***	Infrared
Spectral Region	IR	***X Axis***	Wavenumber (cm-1)
Y Axis	Absorbance	***Spectrum Range***	400.6890 - 3999.4336
Points Count	1932	***Data Spacing***	1.8637

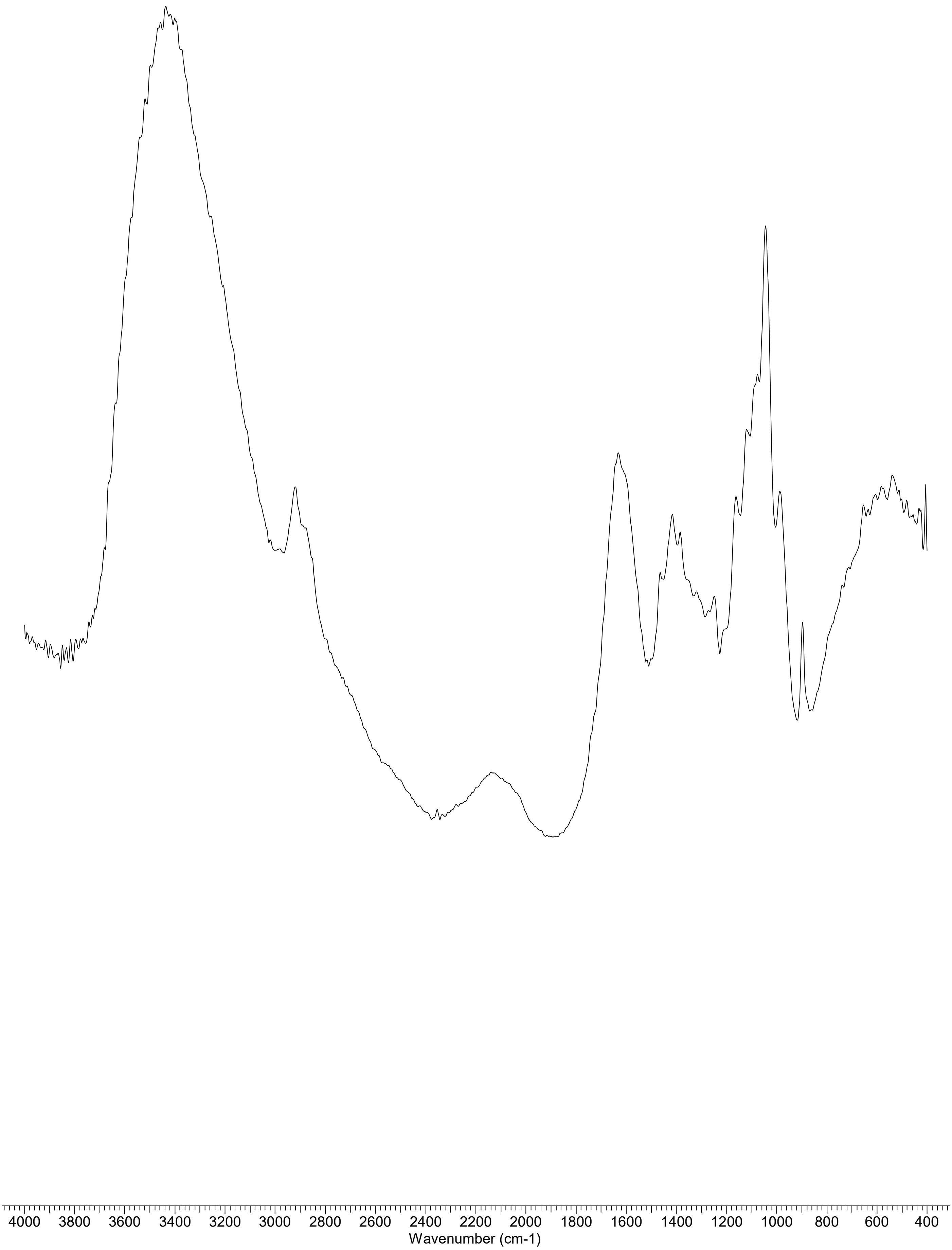

Supplement: Supplementary file 1 [file polymers-15-04597-s001.zip › Supplementary File S3 FTIR data.pdf]
